# NAD-Biosynthetic and Consuming Enzymes as Central Players of Metabolic Regulation of Innate and Adaptive Immune Responses in Cancer

**DOI:** 10.3389/fimmu.2019.01720

**Published:** 2019-07-25

**Authors:** Valentina Audrito, Antonella Managò, Federica Gaudino, Leonardo Sorci, Vincenzo Gianluca Messana, Nadia Raffaelli, Silvia Deaglio

**Affiliations:** ^1^Department of Medical Sciences, University of Turin, Turin, Italy; ^2^Italian Institute for Genomic Medicine, Turin, Italy; ^3^Division of Bioinformatics and Biochemistry, Department of Materials, Environmental Sciences and Urban Planning, Polytechnic University of Marche, Ancona, Italy; ^4^Department of Agricultural, Food and Environmental Sciences, Polytechnic University of Marche, Ancona, Italy

**Keywords:** immunometabolism, metabolic reprogramming, immune cell regulation, NAD, tumor microenvironment

## Abstract

Cancer cells, particularly in solid tumors, are surrounded by non-neoplastic elements, including endothelial and stromal cells, as well as cells of immune origin, which can support tumor growth by providing the right conditions. On the other hand, local hypoxia, and lack of nutrients induce tumor cells to reprogram their metabolism in order to survive, proliferate, and disseminate: the same conditions are also responsible for building a tumor-suppressive microenvironment. In addition to tumor cells, it is now well-recognized that metabolic rewiring occurs in all cellular components of the tumor microenvironment, affecting epigenetic regulation of gene expression and influencing differentiation/proliferation decisions of these cells. Nicotinamide adenine dinucleotide (NAD) is an essential co-factor for energy transduction in metabolic processes. It is also a key component of signaling pathways, through the regulation of NAD-consuming enzymes, including sirtuins and PARPs, which can affect DNA plasticity and accessibility. In addition, both NAD-biosynthetic and NAD-consuming enzymes can be present in the extracellular environment, adding a new layer of complexity to the system. In this review we will discuss the role of the “NADome” in the metabolic cross-talk between cancer and infiltrating immune cells, contributing to cancer growth and immune evasion, with an eye to therapeutic implications.

## Composition of the Tumor Microenvironment: Supportive and Immunoregulatory Cells

**Figure 1:**
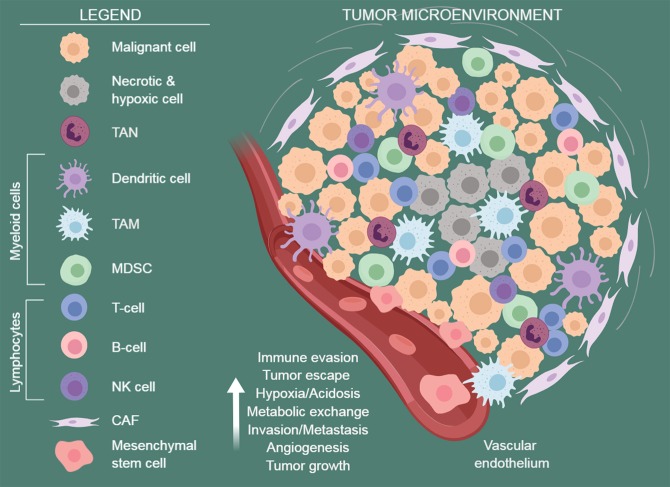
The tumor microenvironment. A schematic view of the tumor microenvironment components. Established cancers are usually surrounded by a wide array of stromal cells and infiltrating immune cells of both innate and acquired immunity, such as MDSCs, macrophages, dendritic cells, neutrophils, NK cells, and lymphocytes. They form a complex regulatory network that supports tumor growth by creating a tolerogenic environment that enables cancers to evade immune surveillance and destruction. TAN, tumor-associated neutrophils; TAM, tumor-associated macrophages; MDSC, myeloid-derived suppressive cells; CAF, cancer-associated fibroblasts. Figure arrange using BioRender software https://biorender.com/.

The solid tumor microenvironment (TME), as well as the lymphoid niche, is a dynamic and multicellular ecosystem with complex interactions (1, 2). Intercellular crosstalk within this niche is driven by multiple receptor-ligand systems, as well as by locally synthesized soluble proteins, including chemokines/cytokines, interleukins, interferons, growth, and angiogenic factors (3, 4). This unique environment is essential for tumor growth, metastatic dissemination, and drug-resistance. Furthermore, the cellular and soluble components of the TME have an important role in shaping metabolic reprogramming of cancer cells, an established hallmark of cancer, and in creating an immunosuppressive environment (5–8), as showed in Figure 1.

The formation of the TME and the regulation of immune responses are orchestrated by different types of host cells, including endothelial cells (ECs), mesenchymal stem/stromal cells [MSCs, including cancer-associated fibroblasts (CAFs) and tumor-associated MSCs (TA-MSCs)], and tumor-infiltrating immune cells [i.e., tumor-infiltrating lymphocytes (TILs), tumor-associated macrophages (TAMs), myeloid-derived suppressor cells (MDSCs), and tumor-associated neutrophils (TANs)]. Their concerted action promotes tumor growth and spreading (1, 2, 9, 10) (Figure 1).

### Endothelial Cells (ECs)

ECs support blood supply, nutrient transport, metabolic homeostasis, and immune cell trafficking, and are involved in inflammatory response (11).

To provide nutrients to the growing tumor, ECs form tumor-associated (angiogenic) vessels originating from locally pre-existing vessels or recruiting bone marrow-derived endothelial progenitors. They also represent the first interface between circulating blood cells, tumor cells, and the extracellular matrix, thereby playing a central role in regulating leukocyte recruitment, tumor cell features, and metastasis dissemination (12). Tumor-associated EC are dysfunctional, partly as a consequence of local hypoxia, which induces the production of soluble factors promoting neo-angiogenesis and contributing to tumor dissemination and chemoresistance (13, 14). Among these factors, vascular endothelial growth factor A (VEGF-A) can also play a critical role in the control of immune tolerance, linking immune suppression with angiogenesis (15).

### Mesenchymal Stem/Stromal Cells (MSCs)

MSCs strongly affect the development and progression of various cancers (16). Stromal cells represent the main cell component with both supportive and immunoregulatory functions; they derived from multipotent cells of mesodermal origin which virtually reside in all tissues with an important role in tissue regeneration (16). MSCs have been found to migrate to tumors and to evolve into TA-MSCs and CAFs with an active role in tumor survival, proliferation, migration and drug resistance, and therefore, recently emerged as attractive targets or tools for anticancer approaches (17, 18).

CAFs are the most abundant resident cells of the TME. Numerous studies have demonstrated that CAFs have prominent roles in cancer pathogenesis (19, 20). Mechanistically, CAFs shape the extracellular matrix (ECM) structure, which supports the tumor cells (i) to invade and interact with stromal cells through the secretion of growth factors, cytokines and chemokines including interleukin-6 (IL-6), transforming growth factor-β (TGF-β) and CC-chemokine ligand 2 (CCL2); (ii) to amplify immune evasion recruiting immune cells, especially immunosuppressive cells into the tumor stroma; (iii) to promote the establishment of an intratumoral vascular network through proinflammatory and proangiogenic mediators (21). CAFs also activate epithelial-mesenchymal transition (EMT) in cancer cells, conferring their pro-invasive and stem-like features (22). In addition, CAFs are plastic cells that co-evolve with cancer cells and acquire a pro-tumor phenotype, contributing to tumor evolution (23). Due to the pro-tumor role of CAFs in support cancer development they become promising therapeutic targets for cancer therapy (21).

### Tumor-Infiltrating Lymphocytes (TILs)

TILs are additional immune components, crucial in driving immune responses within the TME, adding more complexity in the composition of the TME (3). TILs are white blood cells, including T and B cells, that have left the bloodstream and migrated toward a tumor or tissue resident (1, 24). Their abundance varies according to tumor type and stage and in some cases relates to disease prognosis, tumor progression, and response to anticancer therapy (1, 25, 26). T cell differentiation status, survival, activation or “stemness properties” are determining factors of antitumor potency (27) and functions of TILs dynamically change within the TME (28). Sometimes TILs, specifically cytotoxic CD8^+^ memory T cells and CD4^+^ T helper 1 (Th1), which are normally antigen “experienced,” kill tumor cells (29), and the presence of lymphocytes in tumors is often associated with a better prognosis during immunotherapy treatment, including the adoptive transfer of naturally- TIL or genetically-engineered T cells and the use of immune-checkpoint inhibitors (26, 30). However, very often, during cancer progression and chronic inflammation, T cells become exhausted due to the persistent antigen exposure. T cell exhaustion is a state of T cell dysfunction defined by poor effector function, sustained expression of inhibitory receptors, such as programmed cell death protein 1 (PD1) and cytotoxic T lymphocyte antigen 4 (CTLA4), and transcriptional programs altered compared with functional effector or memory T cells (31).

Regulatory T (Treg) cells are another TME cell type that has immunosuppressive functions in cancer, inhibiting recognition, and clearance of tumor cells by the immune system (30, 32, 33). Tregs are characterized by the expression of CD4, CD25, and forkhead box P3 (FOXP3) as their master regulator. Foxp^3^ Treg can originate in the thymus (naturally occurring Treg) or can be induced (iTreg) in the periphery by soluble cytokines and cell-cell contact (34) and are essential for maintaining peripheral tolerance and limiting auto-immune diseases. However, the proportions of Tregs are much higher in the circulation of patients with solid and hematologic malignancies and accumulation of Tregs in the tumor microenvironment is associated with disease progression and reduced survival (35, 36). From a functional point of view, Tregs inhibit both cellular and humoral immune responses by suppressing expansion and activation of conventional CD4^+^ and cytotoxic CD8^+^ T cells, and natural killer cells, mainly through the secretion of suppressive cytokines, such as TGF-β and IL-10. The development of agents that specifically inhibit Treg functions or remove them from the TME will permit new approaches for anticancer immunotherapy (37).

### Tumor-Associated Macrophages (TAMs)

TAMs are important mediators of tumorigenesis, resident in the tissue or deriving from peripheral reservoirs such as the bone marrow (BM) and spleen (2). Macrophages are functionally plastic and can be polarized into the immune stimulating and antitumor M1 subtype, or into “alternatively activated” M2 macrophages producing type II cytokines, promoting anti-inflammatory responses, and having pro-tumorigenic functions (38, 39). Macrophage polarization is finely tuned in response to different microenvironmental stimuli (40). For example, hypoxia may mediate this transition from tumor suppressing to tumor promoting macrophages (41). Furthermore, it has been shown a reciprocal regulation between CAFs and M2 macrophages: CAFs promote monocyte recruitment and polarization toward the M2 phenotype, leading to the enhancement of proangiogenic features, in parallel M2 macrophages are able to induce fibroblast activation (42). It is well-known that TAMs have a clear role in supporting multiple aspects of tumor progression (43). For example, TAMs promote tumor cell invasion through a paracrine loop that involves tumor-derived colony-stimulating factor 1 (CSF-1) and macrophage-derived epidermal growth factor (EGF) (43, 44). Moreover, TAMs induce immune suppression [reviewed in (45)] mediated by (i) expression of inhibitory receptors, including human leukocyte antigens (HLA)-E and HLA-G and T cell immune checkpoint ligands, such as PDL1, PDL2, CD80 and CD86, which directly inhibit T cell functions and NK cells; (ii) release of several cytokines, such as IL-10 and transforming growth factor-β (TGFβ), that contribute to feed a strong immunosuppressive microenvironment by inhibiting CD4^+^ (Th1 and Th2 cells) and CD8^+^ T cells and inducing Treg cell expansion and recruitment through CCL2, CCL3, and CCL20. Lastly, they induce depletion of essential amminoacids for cytotoxic activity of T cells including l-arginine and tryptophan, or production of kynurenine by indoleamine 2,3-dioxygenase (IDO) that inhibits T cell cytotoxicity.

Reversion of TAMs back to an M1 phenotype has also been reported (46), highlighting a potential therapeutic opportunity in which re-education of TME-resident macrophages might have beneficial anti-tumorigenic effects (45).

### Myeloid-Derived Suppressor Cells (MDSCs)

Along with TAMs, MDSCs are considered major promoters of tumor immune evasion (47). This population of myeloid cells, functionally defined as immunosuppressive, arises as a consequence of aberrant myelopoiesis typical of cancer (48). During tumorigenesis, MDSCs are mobilized from BM, via CXCR4/CXCL12 axis (49) and infiltrate tumors, where they promote tumor neoangiogenesis, producing endothelial growth factors [e.g., VEGF, basic fibroblast growth factor (bFGF)] (47). At the same time, they disrupt the major mechanisms of immunosurveillance, including antigen presentation by dendritic cells (DCs), T cell activation, M1 macrophage polarization and NK cell cytotoxicity, as reviewed in Safari et al. (50) and Wang et al. (51). Pharmacological inhibitors of CXCR4, are now under clinical investigation for the mobilization of immune and hematopoietic stem cells (52). Noteworthy, depletion of MDSCs by chemotherapeutic agents (e.g., gemcitabine, cyclophosphamide) can efficiently contribute to their anticancer action (48, 50, 53).

### Tumor-Associated Neutrophils (TANs)

More recently, a population of neutrophils, known as TANs, has been identified as tumor supporter promoting growth, invasion, and angiogenesis of cancer cells, although they have been classically considered to exhibit a defensive response against tumor cells. Like all other leukocytes, they migrate into tissues under the effect of specific chemokines, cytokines and cell adhesion molecules for example TGF-β and IL-8 induce the formation of a pro-tumorigenic (N2) phenotype capable of supporting tumor growth and suppressing the antitumor immune responses (54, 55). Accordingly, TGF-β blocking results in the recruitment and activation of TAN with an anti-tumor phenotype (54). The main tumor-promoting mechanisms of TANs include secretion of chemokines and/or cytokines, reactive oxygen species (ROS), and matrix-degrading proteinases, among others, conditioning tumor immune surveillance, metastasis, invasion, angiogenesis, and cellular proliferation (55, 56).

## Tumor-Stroma Metabolic Cross-Talk in TME

It has been shown that the environment surrounding tumor cells is characterized by low oxygen tension (i.e., hypoxia) due to the abnormal blood vessel formation, defective blood perfusion, and unlimited cancer cell proliferation (14). The progression of hypoxia over time is a consequence of increased oxygen consumption and high glycolytic rate of aberrantly proliferating cancer cells (aerobic glycolysis or Warburg metabolism), leading to lactate dehydrogenase (LDH) activity, lactate excretion and TME acidosis, which alters the tumor-stroma “metabolic cross-talk” (Figure 1). *Vice versa*, hypoxia rapidly fosters energy production in tumor cells via glycolysis through hypoxia-inducible factor 1-alpha (HIF-1α)-mediated transcriptional control (57, 58). In addition, a hypoxic environment also modulates tumor-associated immune and stromal cells metabolism and fate. The rapid consumption of extracellular glucose and glutamine by tumor cells, especially in hypoxic conditions, leads to the accumulation of extracellular lactate, which was shown to affect several cell types within the TME (59). Increased lactate levels promote the insurance of an immune-permissive microenvironment by attenuating DCs and T cell activation, monocyte migration, and polarization of resident macrophages to TAMs (60–63). Furthermore, lactate accumulation promotes angiogenesis, stabilizes HIF-1α and activates NF-kB and PI-3 kinase signaling in endothelial cells, as well as inducing secretion of the proangiogenic factor VEGF from tumor-associated stromal cells (64–66). The secretion of lactate via the monocarboxylate transporter (MCT3) is coupled to the cotransport of H^+^, which supports acidification of the cellular microenvironment (59). The surplus of CO_2_ generated in mitochondrial decarboxylation reactions contributes to extracellular acidification as well (67). Then, a class of extracellular carbonic anhydrases (CA) can convert CO_2_ to H^+^ and HCO3^−^. Accordingly, expression of CAIX isoforms is elevated during hypoxia and can be considered a proxy for HIF-1α signaling (68). A consequence of increased extracellular acidification is the stimulation of the proteolytic activity of MMPs that promotes the degradation of the extracellular matrix components enhancing tumor invasion (69).

Lactate in TME can be also recycled, as occurs in the Cori cycle in the liver. In this reciprocal metabolite changes between cancer cells and immune/stromal cells, lactate produced under hypoxic conditions by glycolytic cells can be re-uptaken by aerobic cells, via MCT1, and utilized for mitochondrial tricarboxylic acid (TCA) cycle and oxidative phosphorylation (OXPHOS) (70, 71). This well characterized mechanism is known as the “reverse Warburg effect” (70, 72). In a model of epithelial cancer, tumor cells instruct the normal stroma to transform into a wound-healing stroma, providing the necessary energy-rich microenvironment for facilitating tumor growth and angiogenesis (72, 73). This metabolic cross-talk is evident in breast, prostate and ovarian cancer (74–76).

Both innate and adaptive immune cells increase their metabolic capacity upon stimulation, promoting energy generation, and biosynthesis supporting proliferation, effector molecule production, and differentiation (77). The impact of such altered metabolic state and levels of metabolites in TME on immune cell function is emerging. For example, a competition between tumor cells and T cells for the glucose pool in the aerobic microenvironment is linked to suppressed effector T-cell functions. In fact, activated T cells rely on glucose metabolism, up-regulating GLUT1 transporter via T cell receptor (TCR) and CD28-induced Akt activation (78, 79). Critical concentrations and/or lack of two amino acids, glutamine and arginine, necessary for T-cell activation, differentiation and proliferation, are therefore inhibitory to T cell functions (79).

The TME shows high levels of immunosuppressive metabolic byproducts, including a turnover in the TME release of adenosine triphosphate (ATP) and nicotinamide dinucleotide (NAD) which are metabolized by the ectoenzymes CD39, CD73, and the NADase CD38 to adenosine (80, 81). Adenosine binds to the T-cell adenosine A2R receptor inhibiting effector T-cell functions and stimulating Treg cells (82, 83). Furthermore, the adenosinergic axis is over-functional in hypoxic conditions, connecting adenosine-mediated immunesuppression to low oxygen tension (84, 85).

Overall, a better understanding of the critical players within the TME and their specific roles in immune regulation will help design of metabolism-targeted therapeutic strategies for improving immunotherapy regimens in cancer.

Recently, NAD pathway enzymes and metabolites were shown to affect immune-cell functions and fate and alter the cancer cell-TME crosstalk. The following paragraphs are focused on describing these molecular circuits and their therapeutic implications.

## NAD Homeostasis: An Overview

**Figure 2:**
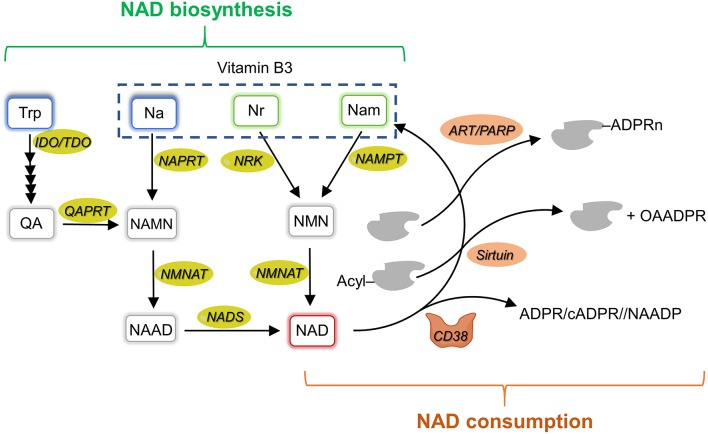
NAD metabolism overview. Schematic representation of mammalian NAD metabolism including biosynthetic (left side, in green) and consuming (right side, in orange) pathways. Na, nicotinic acid; NAD, nicotinamide adenine dinucleotide; NAPRT, nicotinate phosphoribosyltransferase; NAMN, nicotinate mononucleotide; NAAD, nicotinate adenine dinucleotide; Nam, nicotinamide; NAMPT, nicotinamide phosphoribosyltransferase; NADS, NAD synthetase; NMN, nicotinamide mononucleotide; NMNAT, NMN adenylyltransferase; Nr, nicotinamide riboside; NRK, nicotinamide riboside kinase; QA, quinolinic acid; QAPRT, quinolinate phosphoribosyltransferase; IDO, indoleamine 2,3-dioxygenase; TDO, tryptophan 2,3-dioxygenase; Trp, tryptophan; OAADPR, 2'-O-acetyl-ADP ribose; ART, ADP-ribosyltransferases; PARP, poly-ADP-ribose polymerase; ADPR, ADP-ribose; cADPR, cyclic ADPR; NAADP, nicotinic acid adenine dinucleotide phosphate.

NAD is a vital molecule governing many metabolic processes. It is used as a redox coenzyme by several dehydrogenases, and as a co-substrate by various NAD-consuming enzymes (86, 87). Among them are (i) mono- or poly-ADP ribosyltransferases (including ARTs and PARPs), which transfer the ADP ribose moiety to acceptor proteins resulting in their modification and function regulation, (ii) sirtuins, which catalyze the NAD-dependent deacetylation of metabolic enzymes and transcription factors, thus controlling their activity; (iii) NAD glycohydrolase that generates different NAD metabolites, including ADP ribose (ADPR), cyclic ADP ribose (cADPR) and nicotinic acid adenine dinucleotide phosphate (NAADP), with calcium (Ca^+2^) mobilizing activity. These enzymes are involved in the control of a wide range of biological processes, including transcription, DNA repair, cell adaptation to stress signals, and immune response (88). By catalyzing their reactions, they render NAD continuous re-synthesis an indispensable process. Various NAD biosynthetic routes guarantee the coenzyme regeneration, in different combination and with different efficiency depending on the cell-type and metabolic status (89, 90). A schematic overview of NAD homeostasis is shown in Figure 2 and reviewed in Sharif et al. (87), Magni et al. (91), and Houtkooper et al. (92).

The route which recycles nicotinamide (Nam), produced by the breakage of the N-glyosidic bond in the various NAD-consuming reactions, back to NAD that is considered the major pathway ensuring NAD homeostasis. It involves the phosphoribosylation of Nam to nicotinamide mononucleotide (NMN) by the enzyme Nam phosphoribosyltransferase (NAMPT) and the subsequent adenylation of NMN to NAD by NMN adenylyltransferase (NMNATs). This same route also salvages extracellular Nam that can be of dietary origin or can be formed in the extracellular space by the NAD glycohydrolase activity of the CD38 ectoenzyme acting on extracellular NAD and/or NMN. NAD can also be synthetized from exogenous nicotinamide riboside (NR) and nicotinic acid (NA) through distinct routes that are initiated by NR kinase (NRK) and NA phosphoribosyltransferase (NAPRT), respectively. The former enzyme phosphorylates NR to NMN, whereas the latter enzyme phosphoribosylates NA to nicotinate mononucleotide (NAMN). NMNATs convert NMN to NAD, and NAMN to nicotinate adenine dinucleotide (NAAD). NAAD is finally amidated to NAD by the enzyme NAD synthetase. A *de novo* biosynthetic route, which starts from tryptophan and enters the amidated route from NA, is also operative in several tissues and cell-types. The first and rate- limiting step in this pathway is the conversion of tryptophan to N-formylkynurenine by either IDO or tryptophan 2,3 -dioxygenase (TDO). Four reactions are then required to transform N-formylkynurenine to an unstable intermediate, α-amino-β-carboxymuconate-ε-semialdehyde (ACMS), which undergoes either decarboxylation, directed toward oxidation, or spontaneous cyclization to quinolinic acid (QA) directed toward NAD formation. Indeed, QA is phosphoribosylated to NAMN by the enzyme QA phosphoribosyltransferase (QAPRT), and the formed NAMN enters the NA salvage pathway. Among the enzymes involved in NAD homeostasis, NAMPT, CD38, sirtuins, and IDO are overexpressed in different types of cancer (93) and have been shown to play a role in cancer immune tolerance (94, 95). In the following sections, we will review what is known about their expression and function in the TME.

## NAMPT in Metabolic Regulation and Activation of Myeloid Cells

As the first and rate-limiting enzyme, NAMPT plays a pivotal role in the biosynthesis pathway of NAD from its nicotinamide precursor. It converts Nam and 5-phosphoribosyl-1-pyrophosphate (PRPP) into NMN in a complex reaction that can be significantly improved by a non-stoichiometric ATP hydrolysis (96). NAMPT is found both intracellularly and extracellularly (97, 98). Intracellular NAMPT (iNAMPT) is primarily located in the nucleus and cytosol. Previous studies reported NAMPT in mitochondria as well (99), but this remains a controversial finding (100, 101). As one of the main regulators of NAD intracellular level, NAMPT plays a crucial role in cellular metabolism (102). Conversely, the extracellular form of NAMPT (eNAMPT) has emerged as an important mediator of inflammatory programs (103). eNAMPT has been found in plasma and other extracellular fluids, including the supernatants of numerous cell types (103); however, while the mechanisms behind eNAMPT secretion remain unknown, they do not seem to rely on the classic pathway (104). Notably, the cytokine-like functions appear independent of the protein catalytic activity (105). In keeping with this view, NAMPT's substrates PRPP and ATP are apparently unavailable in the extracellular space to sustain the enzymatic activity (106).

eNAMPT was originally found to be secreted by activated lymphocytes and bone marrow stromal cells by Samal et al. (107) and called pre-B-cell colony enhancing factor [PBEF (107). In 2005, Fukuhara (108) identified eNAMPT as an adipokine and called it visfatin. These different names reflect its role in immune system and adipose tissue regulation.

Independent studies have conclusively shown that NAMPT expression and secretion can be induced by inflammatory signals in immune cells, in particular neutrophils, monocytes and macrophages (109). Both pathogen-derived lipopolysaccharide (LPS) and host-derived inflammatory stimuli, including tumor necrosis factor-α (TNF-α), IL-1β, IL-6, and leptin, can up-regulate *NAMPT* transcription in macrophages and other several types of cells (110–113). Several studies showed stimulation of cytokine release after exposure of cells to exogenous NAMPT, highlighting a role of eNAMPT as an inflammatory mediator as reviewed in Garten et al. (103). Following NAMPT treatment, IL-1β, IL-6, TNF-α, and IL-10 are up-regulated in peripheral blood mononuclear cells (PBMCs) and CD14^+^ monocytes (114). Co-stimulatory molecules such as CD54, CD40, and CD80 are also up-regulated in response to NAMPT treatment, an effect mediated through PI3-kinase and MAPKs p38, MEK1, and JNK (114). Furthermore, in macrophages NAMPT increases MMPs expression and activity (115). *In vitro*, eNAMPT promotes cell survival in macrophages subjected to endoplasmic reticulum (ER) stress, a frequent event in obesity and obesity-associated diseases. eNAMPT induces IL-6 secretion, followed by IL-6-mediated autocrine/paracrine activation of the prosurvival signal transducer STAT3, with a mechanism that is independent of the enzymatic activity (112).

Emerging evidence supports a role of NAMPT in regulating the differentiation program and the metabolic adaptation of myeloid cells. As described previously, activated macrophages can be divided in two subgroups *in vitro*: those with pro-inflammatory activity (M1) involved in first line of defense against bacterial infection, and those with anti-inflammatory activity (M2) that regulate tissue repair and wound healing (116), even if this is an oversimplification of the functional diversity occurring *in vivo*. Metabolic reprogramming of immune cells is required for both pro- and anti-inflammatory responses and a vast spectrum of metabolic statuses accompanies the complexity of phenotypes [reviewed in (117, 118)]. In general, an increase in glycolysis and in glucose uptake is typically associated to an M1 phenotype (119), while M2 macrophages rely on intact TCA cycle and OXPHOS as major source of ATP via electron transport chain and ATP synthase (120, 121). However, in addition to an augmented mitochondrial metabolism, alternatively activated macrophages can also use glycolysis when OXPHOS is disrupted (122). Another important pathway is the pentose phosphate pathway (PPP), which generates pentoses, 5-ribose phosphate and nicotinamide adenine dinucleotide phosphate (NADPH). NADPH is essential in activated M1 macrophages because it fuels ROS production by NADPH oxidase (123), even if other groups demonstrated that NADPH and NADPH oxidase play a role even in M2 differentiation (124). Concerning lipid metabolism, fatty acid synthesis is coupled to pro-inflammatory activity of macrophages, while beta-oxidation is typical of anti-inflammatory macrophages (117).

The increase of glycolysis associated with M1 activation of macrophages is orchestrated by the transcription factor HIF-1α. When cells experience low oxygen levels HIF-1α is stabilized and, upon binding of the HIF-1β subunit, initiates the transcription of genes such as glucose transporter and glycolytic enzymes (125, 126). NF-kB is required for transcriptional activation of HIF-1α (127); whereas, in M2 macrophages, genes involved in metabolic reprogramming are largely controlled by STAT6 and peroxisome proliferator-activated receptor gamma coactivator-1 beta (PGC-1β) (128).

**Figure 3:**
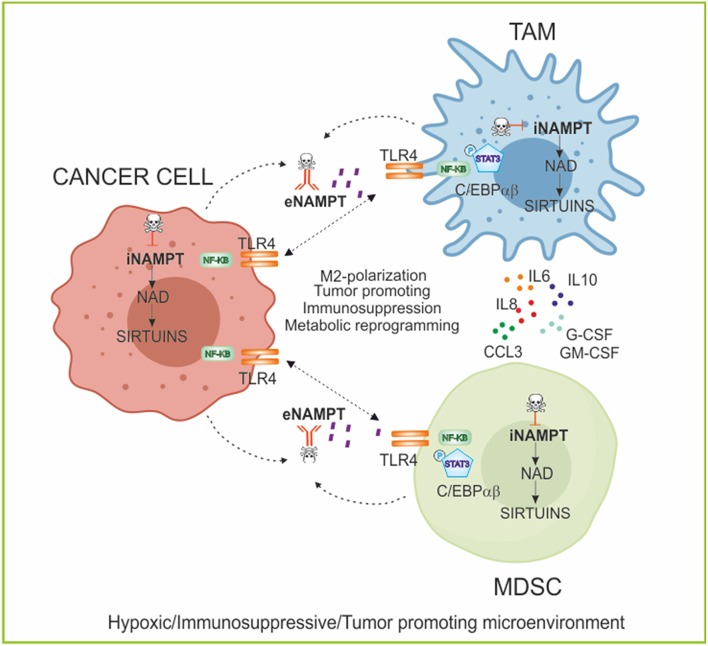
NAMPT in regulating myeloid cell fate and immunometabolism. Role of iNAMPT/eNAMPT in skewing myeloid populations into tumor-supporting M2-like macrophages and myeloid suppressive cells. Specifically, the iNAMPT/sirtuins axis regulates the metabolic reprogramming of cancer and myeloid cells in condition of low oxygen tension; while eNAMPT/TLR4 axis activates intracellular signaling promoting differentiation of myeloid cells and secretion of anti-inflammatory and pro-tumor cytokines creating an immunosuppressive microenvironment. The block of NAMPT functions, using iNAMPT pharmacological inhibitors and/or neutralizing antibodies, can repolarize the myeloid populations and inhibit tumor growth. TLR4, Toll-like receptor 4; C/EBPα/β, CCAAT/enhancer-binding protein α/β; G-CSF, Granulocyte Colony-Stimulating Factor; GM-CSF, Granulocytes-Macrophage Colony-Stimulating Factor; TAM, tumor-associated macrophages; MDSC, myeloid-derived suppressive cells.

Both iNAMPT and eNAMPT influence fundamental monocyte/macrophages processes such as differentiation, polarization and migration, even if the exact role of iNAMPT/eNAMPT in the process of myelopoiesis is incompletely elucidated so far (129–131) as summarized in Figure 3. For example, NAMPT has a role in the induction of an immunosuppressive and tumor-promoting microenvironment in chronic lymphocytic leukemia, where eNAMPT is important for the differentiation of monocytes toward tumor-supporting immunosuppresive M2 macrophage, promoting their differentiation, and polarization in tumor-supportive cells including TAMs (130). Recently, it was demonstrated that iNAMPT acts also on MDSCs, where NAMPT inhibits *CXCR4* transcription, via NAD/SIRT1/HIF-1α axis, and this, in turn, leads to a mobilization of MDSCs and enhances their production of suppressive nitric oxide (132).

Changes in NAD levels characterize different stage of macrophage polarization: in general, higher levels of NAD are typical of classically activated pro-inflammatory macrophages (M1), while NAD levels are lower in alternatively activated anti-inflammatory macrophages (M2). The NAMPT/NAD/SIRT1 axis seems to play a relevant role in myeloid cell functions as shown by the fact that efficient activation of M1 macrophages needs an increase of both NAMPT expression and cytosolic NAD (133). NAMPT-dependent generation of NAD is also crucial in the metabolic switch characterizing the transition from the early initiation phase of acute inflammation, which is anabolic and primarily requires glycolysis, to the later adaptation phase which is catabolic and relies on fatty acid oxidation (FAO) for energy (134). During these processes, also NAD-consuming deacetylases enzymes SIRT1 and SIRT6 have a role in regulating metabolism, increasing fatty oxidation and reducing glycolysis, respectively, coupling metabolic polarity with the inflammatory response, as described with more details later (135, 136). These data support the notion that NAD homeostasis has a crucial role in connecting bioenergetics and inflammation (134). A further feedback loop that links NAD to polarization of myeloid component has been suggested in monocytes, where NAMPT expression is induced by TNF-α via HIF-1α. In turn, NAMPT signaling involving NF-kB pathway activates activating protein 1 (AP1), inducing *IL6* and *TNFA* transcription modulating myeloid cell activation (137).

In congenital neutropenia, a disorder in which patients display accumulation of granulocytic progenitors and no mature neutrophils in bone marrow, it has been shown that granulocyte colony-stimulating factor (G-CSF) is effective as it up-regulates NAMPT, which in turn triggers NAD/SIRT1 dependent granulopoiesis via CCAAT/enhancer-binding protein α/β (C/EBPα/β) up-regulation (129). On the contrary, GM-CSF is not effective in congenital neutropenia because it is unable to activate iNAMPT upregulation and NAD/SIRT1 axis (138). Following the induction of myeloid differentiation with G-CSF, the NAD-consuming enzyme SIRT1 deacetylase C/EBPα at position Lys 161 (129, 138). NAMPT inhibition with FK866 led to the dramatic elevation of acetylated C/EBPα levels and reduced amounts of total C/EBPα protein, accompanied by diminished mRNA expression of C/EBPα target genes (G-CSF, G-CSFR, and ELANE). Moreover, treatment of acute myeloid leukemia cell line HL-60 with recombinant NAMPT or transduction of HL-60 cells with NAMPT-expressing lentiviral construct induced myeloid differentiation of these cells *per sé* (138).

An open question is whether the cytokine-like actions that eNAMPT exerts on myeloid cells are related to its enzymatic activity or are mediated by the binding to a cell surface receptor. The fact that treatment with low concentrations of recombinant eNAMPT is sufficient to activate specific intracellular signaling pathways suggests that eNAMPT has cytokine-like properties and binds to and activates a cell surface receptor. In 2015, Camp et al. identified eNAMPT as a new ligand of the Toll-like receptor 4 (TLR4) (105). The authors demonstrated that in human lung endothelial cells, eNAMPT activates an inflammatory response via activation of NF-kB signaling pathway by binding TLR4-MD2 (105). However, the fact that recombinant eNAMPT is often produced in *E. Coli* strains renders the interpretation of these results controversial for the possible contamination of LPS, the natural ligand of TLR4, and activator of inflammatory programs. New studies have to confirm the TLR4 engagement by eNAMPT and correlate this with myeloid differentiation and plasticity.

The evidence linking myeloid cell fate and NAD/NAMPT could open the way to pharmacological inhibition of either iNAMPT and/or eNAMPT for re-education of myeloid cells. This could be useful in the context of acute inflammation, but also in cancer to force a reversion of immunosuppressive microenvironment, in combination with immunotherapy, as summarized in Figure 3.

For iNAMPT specific small molecules inhibitors exist, most known FK866 (also known as APO866) and GMX1778 (also known as CHS-828), among others (Table 1) (139–143, 159–161). However, most of the data on these drugs describe their effect on the tumor itself, and not on cells of the microenvironment (141, 161). Whether these inhibitors could also affect also eNAMPT activity is unknown, even if, as mentioned before, the enzymatic activity of eNAMPT is controversial. On the other hand, for eNAMPT, the group of Garcia, in order to block only the cytokine-like activity of eNAMPT, has devised a polyclonal eNAMPT neutralizing antibody (130, 144), that could be useful in those condition in which only the extracellular form of eNAMPT is detrimental and intracellular enzymatic activity needs to be preserved.

**Table 1:** Pharmacologic tools currently undergoing pre- or clinical evaluation to block NADome enzymes.

**Agent**	**Mechanism of action**	**Indication**	**Trial Stage**	**References**
**NAMPT INHIBITORS**				
APO866 (FK866)	NAMPTi	T/IC	Clinical phase I	(139)
CHS-828 (GMX 1778)	NAMPTi	T/IC	Clinical phase I	(140)
GNE-617, GNE-618	NAMPTi	T	Pre-clinical	(141)
KPT-9274	Dual NAMPTi/PAX4i	T	Clinical phase I	(142)
OT-82	NAMPTi	T	Clinical phase I	(143)
Blocking antibody	eNAMPT neutralization	T/IC	Pre-clinical	(144)
**CD38 INHIBITORS**				
Daratumumab	Blocking antibody	MM/ALL	Clinical phase III	(145)
Isatuximab	Blocking antibody	MM	Clinical phase II-III	(146)
MOR202	Blocking antibody	MM	Clinical phase II	(147)
Apigenin	CD38i	MD	Pre-clinical	(148)
**SIRTUINS INHIBITORS**				
Cambinol	SIRT1/2i	T/ND	Pre-clinical	(149)
Sirtinol	SIRT1/2i	T/ND	Pre-clinical	(150)
Selermide	SIRT1/2i	T/ND	Pre-clinical	(151)
Tenovins	SIRT1i	T/ND	Pre-clinical	(152)
EX-527	SIRT1i	T/ND	Pre-clinical	(153)
Nicotinamide	SIRTi/NAD precursor	T/ND	Pre-clinical, phase I-II	(154)
**IDO INHIBITORS**				
Indoximod	IDOi	T	Clinical phase I-II	(155)
Epacadostat (INCB024360)	IDOi	T	Clinical phase II-III	(156)
Navoximod	IDOi	T	Clinical phase I	(157)
BMS-986205	IDOi	T	Clinical phase I-II	(158)

*I, inhibitor; T, solid and/or hematological tumors; IC, inflammatory conditions; MM, multiple myeloma; ALL, acute lymphoblastic leukemia; MD, metabolic diseases; ND, neurodegenerative diseases*.

## CD38 in Metabolic Dynamics of T Cells Activation

Cluster of differentiation (CD) protein CD38, first identified as a lymphocyte antigen, is a cell surface glycohydrolase that cleaves a glycosidic bond within NAD to yield Nam, ADP-ribose (ADPR), and cyclic ADPR (cADPR), and converts NAD phosphate (NADP) to NAADP, all calcium (Ca^2+^) mobilizing molecules (162, 163). These molecules bind specific receptors, like the ryanodine receptor on endoplasmic reticulum, the lysosomal two-pore channel and the plasma membrane calcium channel transient receptor (TRPM2), activating calcium signaling, which in turn affects gene expression, cell cycle control, cell survival, energy metabolism, leukocyte trafficking, and inflammation (87).

CD38 is a transmembrane protein with four different forms, according to the cellular localization (164). The most common form of CD38 has a type II membrane orientation, i.e., with the catalytic domain facing the extracellular space. By contrast, the less abundant type III transmembrane form has its catalytic site facing the inside. Intriguingly, soluble intracellular and extracellular forms of CD38 have also been ascribed (165, 166). CD38 is widely expressed both in immune cell types (bone marrow progenitors, natural killer cells, monocytes, and activated T- and B-lymphocytes) and in non-hematopoietic cells (167).

**Figure 4:**
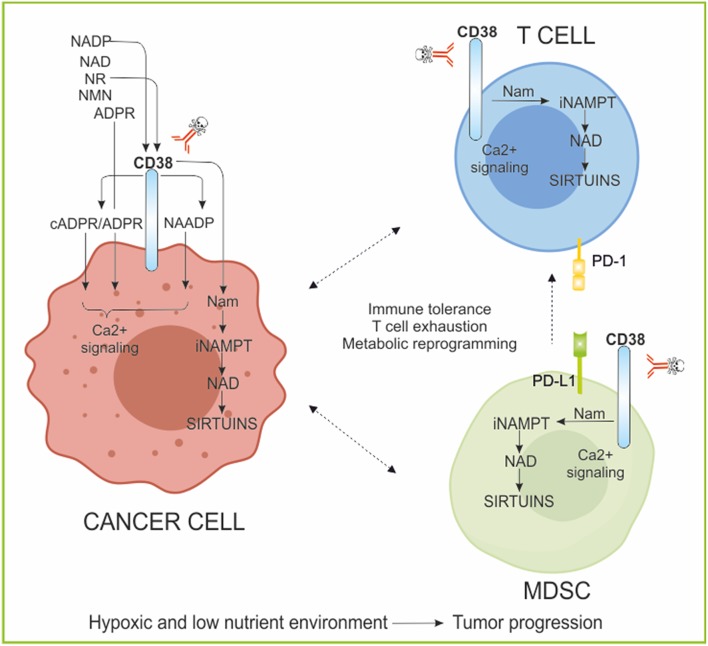
CD38/NAD axis regulates T cell phenotype and responses. The ectoenzyme CD38, expressed by tumor cells and immune cells is involved in the activation of calcium (Ca^2+^) signaling through the generated metabolite cADPR/ADPR/NAADP. Moreover, it metabolizes NAD, releasing Nam, rendering the substrate of NAMPT available for continuous NAD regeneration. These reactions occur also in immune cells modifying NAD concentrations and affecting sirtuins activities. The NAD/CD38/SIRTUINS axis regulates T cell immune cell fate, metabolism, and gene transcription. Evidence of high CD38 expressing immune suppressive cells have been reported in several tumors. CD38 inhibition was sufficient to re-establish T cell proliferation, antitumor cytokine secretion, and killing capability. ADPR, ADP-ribose; cADPR, cyclic ADPR; NADP, NAD phosphate; NAADP, nicotinic acid adenine dinucleotide phosphate; Ca^2+^, calcium; NR, nicotinamide riboside; NMN, nicotinamide mononucleotide; Nam, nicotinamide; MDSC, myeloid-derived suppressive cells; PD-1, programmed cell death protein 1; PD-L1, PD-1 ligand.

CD38 is also an unquestionable contributor to intracellular NAD homeostasis (168, 169) and this apparent “paradox” has been in part reconciled by recent reports demonstrating that CD38 can also degrade circulating NAD precursors such as NMN and NR, thus preventing their fueling of NAD biosynthesis (170, 171). Notably, CD38 enzymatic activity mediates many roles which include metabolism regulation and pathogenesis of heart disease, obesity, aging and inflammation, among the others. Nevertheless, it is well-established that CD38 overexpression is correlated to different hematological malignances including myelomas and leukemias (172). In this contest, a broad immune regulatory role for NAD and CD38 on T cell behavior has been reported (87, 145, 173) and summarized in Figure 4. In order to elucidate the impact of CD38 modulation of NAD homeostasis in T cell, a brief synthesis of T cell metabolism is necessary, as metabolism drives T cell life (36, 174, 175). One of the main challenges of the field in a translational perspective is to manipulate T cell metabolism in order to improve their immune response capacity. Defined metabolic pathways orchestrate T cell development, differentiation, function and persistence (176). TCA/OXPHOS-mediated ATP production is instrumental for the maturation of Naïve T (T_N_) lymphocytes, a population of quiescent non-proliferative cells, in primary lymphoid organs (177). T cell activation is initiated after antigen recognition and TCR ligation. This step, requiring major histocompatibility complex, and co-stimulatory molecules, activates T lymphocytes inducing both a rapid proliferation rate and a differentiation program toward effector functions (176). To sustain both clonal expansion and active immune response, T cells shift to an anabolic metabolism which provides faster ATP production and nutrients supply. While cytolytic CD8^+^ T (Tc) cells dominantly shift metabolism to glycolysis, activated CD4^+^ T helper (Th) cells increase both glycolysis and FAO (178). FAO also supports metabolism of iTreg and long living memory T-cell (Tm) (178). All these T cell subsets, to achieve their metabolic profile, require a coordinated transcriptional program together with a specific system of nutrient uptake. T cells depend on the import of substrates such as glucose, amino-acids (especially glutamine), and glycerol. In T_N_ and Tm cells, increased expression of glucose and glutamine transporters is controlled by the transcription factor c-Myc (36) and regulated by a specific cytokine, IL-7 (175). AKT-mTOR and TLR signaling, as well as the transcription factors HIF-1α, c-Myc and FoxP3, have been shown to directly regulate Treg metabolic programming and development, while HIF-1α and mTOR control the glycolytic phenotype and activation (IFN-γ production) of effector T-cells, Th1, Th2, and Th17 lineages (36). Metabolism underpins T cell cycle through quiescence and activation states and T-cells failure to engage specific metabolic programs is a biological phenomenon accompanying tumor aggressiveness and T cell exhaustions (176). The crosstalk between cancer cell and tumor TILs is played at different levels. As already mentioned, it has been shown that the establishments of nutrients competition between tumor cells and TILs has a primary role in influencing T cell fate and dysfunctions (34, 179–181). Malignant cells push their metabolism toward a Warburg phenotype. The consequent induction of a hypoxic and nutrient-deprived environment (low glucose, glutamine, glycine, and serine) shapes a tumor sustaining microenvironment and immune tolerance (179). Indeed, T cells migrating to tumors sites must adapt to both (i) nutrient-depleted environments (182) and contemporarily to (ii) the presence of hypoxic tumor-derived metabolites including lactate, adenosine, cyclic adenosine monophosphate (cAMP), IDO/kynurenine.

In this context, CD38-mediated Ca^2+^ mobilization can directly affect T cell metabolism. In the physiology of a T lymphocyte, Ca^2+^ controls T cell gene expression and consequently differentiation, development and cytotoxicity (183). Alteration of Ca^2+^ signaling affects immune deregulation and consequently tumor initiation and progression (183–186). A second level of T cell metabolic reprogramming control by the CD38/NAD axis also involves sirtuins (173). Indeed, a lot of literature has been produced on the role of SIRT1, as a key modulator of immune cell functions, as described in a dedicated section of this review (166, 187, 188). In this case, the inverse correlation between expression of CD38 and intracellular NAD contents, act on SIRT1-mediated post-transcriptional control of key genes involved in T cell functions (173). Furthermore, it was recently shown that CD38 is highly expressed by specific subsets of immunosuppressive TILs (i.e., Treg and Th17) (34, 36, 173, 189) and by MDSC, another key immunosuppressive cellular component of tumor milieu represented (190). Both, CD38^high^MDSC cells-mediated suppression of activated T-cells and the concomitant expression of CD38 with exhaustion markers on T cells, for example PD1, pointed to an active role of CD38 in modulating T cell metabolism and fate toward the generation of an immune tolerant landscape in tumor (173). Evidence of high CD38 expressing Treg have been reported for multiple myeloma and acute lymphoblastic leukemia, where the use of mAbs against CD38 (daratumumab, isatuximab, and MOR202, Table 1) is more than a promising therapeutic option to reestablish a functional immune surveillance (145–147, 189, 191, 192). In these tumor models, suppression of CD38^+^ cancer cells associate with an increase in T-helper and cytotoxic T lymphocytes, T-cell functional response and TCR clonality (191, 192). A functional relationship between CD38 and Th17 has also been highlighted (173). Th17 is a CD4^+^ T cell subpopulation secreting IL17, which gained interest in the field of immunotherapy due to their self-renewal, plasticity and hematopoietic stem-like phenotype (173, 193). Adoptive T cell transfer (ACT) therapy is a powerful strategy developed for controlling cancer (194, 195). The emerged staminal potential of Th17, together with their ability to persist for long times at tumor sites, made of this T cell subset an ideal candidate to improve ACT efficacy (173, 196, 197). Chatterjee et al. recently demonstrated that, SIRT1-dependent deacetylation of the transcription factor forkhead box O1 (FOXO1) drives the functional homing in different organs of a hybrid Th1/Th17 population 24 h after ACT. Most importantly, they reported that, the decrease of CD38 expression on Th17 cells leads to the increase of intracellular NAD concentration, reinforcing the SIRT1-dependent immune efficacy of this T cell population (173). For these reasons, the inhibition of CD38 has been proposed not only to specifically target CD38^high^ immune suppressive cell populations (MDSCs, Treg), but also to improve tumor control via ACT therapy or using immunomodulatory drugs (173, 191, 192).

Lastly, very recently CD38 was considered as major acquired mechanism of resistance to PD-1/PD-L1 blockade, causing CD8^+^ T cell suppression. Co-targeting of CD38 and PD-L1 improves anti-tumor immune response. CD38 manipulation was sufficient to regulate CD8^+^ T cell proliferation, antitumor cytokine secretion, and killing capability (198).

## Sirtuins and Epigenetic Regulation of Immune Response

Sirtuins, initially described as transcriptional silencers in yeast (199), represent a class of NAD-dependent enzymes with deacetylase activity. So far, seven isoforms (SIRT1-7) constitute the family of mammalian sirtuins, which differ in subcellular compartmentation, enzymatic activity, and *in vivo* substrate selectivity (200). As a primary cellular location, SIRT1, SIRT6, and SIRT7 are found in the nucleus, SIRT2 in the cytoplasm, and SIRT3-SIRT5 in mitochondria (201). However, recent reports have shown that sirtuins are not anchored to precise subcellular compartments, and may shuttle between them, depending on cell type or physio-pathological conditions (202–205). The canonical reaction catalyzed by sirtuins is the transfer of an acetyl group from protein lysine residues to the ADPR moiety of NAD. As a result, the reaction produces Nam, first released, the deacetylated lysine, and 2'-O-acetyl-ADP ribose (206). Although lysine deacetylation is the primary activity of sirtuins, recent studies have shown that these enzymes can remove a variety of other acyl-lysine groups (207). Some sirtuins act as ADP-ribosyltransferases, although the biological relevance of such activity is incompletely understood. Mammalian sirtuins target different proteins in an isoform-specific fashion (207, 208), allowing their regulation of multiple processes like energy metabolism, epigenetic regulation of gene expression, DNA repair, inflammation, cellular stress resistance, healthy aging, tumorigenesis, autophagy, and apoptosis as reviewed in Haigis and Sinclair (208), Finkel et al. (209), and Houtkooper et al. (210).

Emerging evidence demonstrated that sirtuins are key regulators of inflammatory stress response in immune and non-immune cells (95, 211–213). Sirtuins are involved in epigenetic regulation, through deacetylation of histones and/or non-histone proteins, of metabolic, phenotypic, and bioenergetics reprogramming of immune cells (immuno-metabolism) (210, 212–214).

**Figure 5:**
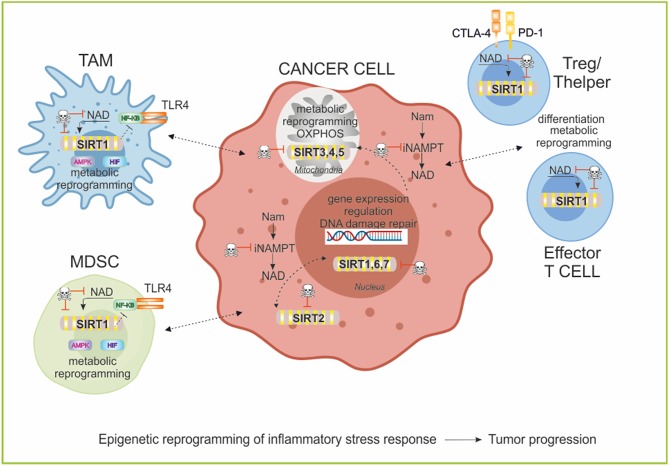
Sirtuins and epigenetic regulation of immune cell functions. Sirtuins are a family of 7 members with different subcellular localization. These NAD-dependent deacetylases are involved in epigenetic regulation of metabolic reprogramming of cancer and immune cells and in promoting T cell differentiation and function. Particularly, in myeloid cells SIRT1 decreases inflammation negatively regulating NF-KB pathway, and inducing a metabolic rewiring mediated by its activity on AMPK/PGC-1α and HIF-1α stabilization. In T cell populations, SIRT1 modifies the phenotypic plasticity of Thelper and Treg and induces T cell tolerance. The manipulation of the Sirtuins/NAD axis is an important area of study for therapeutic implications in cancer research to repolarization of immune cell responses and to block tumor progression. TLR4, Toll-like receptor 4; AMPK, AMP-activated protein kinase; HIF, Hypoxia-inducible factor; PD-1, programmed cell death protein 1; CTLA-4, Cytotoxic T-Lymphocyte Antigen 4; TAM, tumor-associated macrophages; MDSC, myeloid-derived suppressive cells.

SIRT1 is the most extensively studied sirtuins, especially for its role in aging (210, 214). In addition, SIRT1 is involved in controlling stem cell development, cell differentiation and autophagy, metabolic reprogramming and inflammation (209, 215). SIRT1 is also the most studied among sirtuins involved in immune regulation and here we summarized some SIRT1 activities in epigenetic regulation of metabolism and immune response (Figure 5).

Epigenetic mechanisms are essential to the development and differentiation of the immune system, as well as in related pathologies (216–218). Epigenetic mechanisms include multilevel intracellular events that influence chromatin structure and gene expression such as histone methylation and acetylation, as well as DNA methylation, non-coding RNAs and chromatin remodeling (219). Further, numerous signals (i.e., TCR, TLRs, inhibitory receptors, and cytokines) drive changes in the epigenome that result in downstream modulation of immune responses (77). TLR signaling in macrophages regulates differentiation/polarization and activation in response to pathogens affecting gene expression and metabolic reprogramming (220, 221). In particular, TLR4 engagement by LPS in macrophages drives a shift toward a glycolytic metabolism impairing mitochondrial respiration (222), resulting in a marked shifts in NAD/NADH ratios, which influence the activities of SIRT1, potentially altering deacetylation of histone and non-histone substrates (134, 223). Liu et al. found in TLR4-stimulated THP-1 promonocytes that SIRT1 support a switch from increased glycolysis to increased FAO as early inflammation converts to late inflammation (134). The shift to late acute inflammation and elevated FAO required peroxisome proliferator-activated receptor gamma coactivator (PGC-1α),a known target of SIRT1 (187, 224–227). A circuit of AMP-activated protein kinase [(AMPK)/SIRT1/PGC-1α] results in the deacetylation and modulation of the activity of downstream SIRT1 targets that include the PGC-1α and the FOXO1 and FOXO3a transcription factors. The AMPK-induced SIRT1-mediated deacetylation of these targets explains many of the convergent biological effects of these two energy sensors, AMPK and SIRT1, on cellular metabolism (225, 226).

Recent studies have showed that the regulation of innate immunity and energy metabolism are connected through antagonistic crosstalk between NF-κB and SIRT1 signaling pathways (228). NF-κB signaling has a major role in innate immunity defense, while SIRT1 regulates the oxidative respiration and cellular survival (229). However, NF-κB activation can stimulate glycolysis during acute inflammation, whereas SIRT1 activation inhibits NF-κB signaling and enhances oxidative metabolism and the resolution of inflammation. SIRT1 inhibits NF-κB signaling directly by deacetylating the p65 subunit of NF-κB complex (230). SIRT1 stimulates oxidative energy production via the activation of AMPK, peroxisome proliferator activated receptor (PPARα) and PGC-1α and simultaneously, these factors inhibit NF-κB pathway and suppress inflammation (225, 226, 231). Using a myeloid cell-specific SIRT1 knockout (Mac-SIRT1 KO) mouse model, Schug et al. show that ablation of SIRT1 in macrophages renders NF-κB hyperacetylated, resulting in increased transcription of proinflammatory target genes. Consistent with increased proinflammatory gene expression, Mac-SIRT1 KO mice challenged with a high-fat diet display high levels of activated macrophages in liver and adipose tissue, predisposing the animals to development of systemic insulin resistance and metabolic derangement (232). In some cases, the effects of SIRT1 in regulating metabolism of immune cells are mediated by HIF-1α (233). SIRT1 can bind and deacetylate HIF-1α resulting in a stabilization or in an inhibition of the protein, depending on the cells and context (234, 235). The SIRT1-HIF-1α axis bridges the innate immune signal to an adaptive immune response by directing affecting metabolism, cytokines production, and differentiation of immune cells (236). For example, (i) SIRT1 can limit the function and differentiation of MDSCs through HIF-1α-induced glycolytic metabolic reprogramming (237), (ii) SIRT1 can regulate T helper 9 (Th9) cell differentiation through the mTOR/HIF-1α-dependent glycolytic pathway (238). The interplay between HIFs and sirtuins may also extend to stress settings such as hypoxic tumors, in which cellular redox balance is perturbed (64, 239).

For adaptive immune cells, SIRT1 has a key role in mediating the differentiation of T cell subsets in a NAD-dependent manner. T cells exhibit remarkable phenotypic and functional plasticity during immune responses (240). SIRT1 is involved in (i) Th and Treg cell differentiation (238, 241); (ii) SIRT1 signals in DCs can repress PPARγ activity and promote T helper 2 (Th2) cell responses in airway allergy through metabolism-independent manners (242); (iii) SIRT1 interacts with c- Jun and inhibits CD4 T cells to mediate T cell tolerance (243); (iv) SIRT1 regulates CD8 T-cell differentiation interacting with basic leucine zipper transcription factor ATF-like (BATF) and regulating both epigenetic remodeling and energy metabolism of T cells (244). Furthermore, (v) SIRT1/FOXO1 axis regulates metabolic reprogramming of terminally differentiated memory T cells, as previously described (188).

Finally, SIRT1 has been shown to play also important roles in physiological processes affecting organismal longevity as well as stem cell function and self-renewal (245, 246). In macrophages, SIRT1 is emerging as critical positive modulator of self-renewal, regulating G1/S transition, cell cycle progression and a network of self-renewal genes (247).

Similar functions in regulating inflammation and metabolism are exerted also by SIRT2 and SIRT6 (134, 213, 248).

Interactions of cellular metabolic and epigenetic pathways and how these two key biological processes interplay to feedback modulate immune cell function is attracting in cancer therapy. Sirtuins/NAD axis has proven to be a crucial link between epigenetics and metabolism, and hence, it is an important area of study for therapeutic implications (215). While there are only specific activators or inhibitors for SIRT1 exist, drugs that affect NAD levels or NAD precursors offer the possibility to regulate all seven sirtuins coordinately (239). These compounds can be used alone or in combination with existing cancer therapies. The effects of SIRT1 inhibitors (e.g., cambinol, sirtinol, tenovins, Ex-527, Table 1) are currently studied mainly in the context of cancer (239). Very recent data show the impact of SIRT1 inhibition or genetic deletion on T cell responses, particularly on Treg differentiation. Genetic deletion or pharmacologic inhibition of SIRT1 through EX-527 improves Foxp3^+^ Treg number and function through increased Foxp3 transcription its acetylation, leading to decreased Foxp3 turnover from ubiquitination and poly(ADP)ribosylation. As a result, targeting SIRT1 increases both central and inducible Foxp3^+^ Tregs and promotes their suppressive functions, as summarized in Chadha et al. (249). SIRT1 inhibition is therefore useful in the context of graft-vs.-host disease (GVHD), to extend allograft survival (249–251). However, there are a number of studies in which SIRT1 deletion or inhibition led to proinflammatory conditions, indicating that regulation of the system is still incompletely understood (249, 252). Interestingly, the Nam generated in deacetylase reactions by SIRTs acts as a negative feedback regulator of SIRT activity (253, 254). This Nam is converted back to NAD by the action of NAMPT and NMNATs. Hence, NAD-biosynthetic enzymes, in particular NAMPT, also regulate sirtuins signaling (255) providing the rational to use NAMPT inhibitors to interfere with Sirtuins functions.

Overall these results indicate that sirtuins broadly coordinate innate and adaptive immune reprogramming and represent druggable immunometabolic enhancement targets, useful also to repolarize immune cells in TME.

## Immunosuppression via Tryptophan Catabolism: The Role of Kynurenine Pathway Enzymes

Amino acid catabolism is a key effector in driving immune tolerance. IDO is a cytosolic, heme-dependent enzyme responsible for the rate-limiting step of *de novo* NAD synthesis from tryptophan in extrahepatic tissues. The catalyzed-reaction yields N-formylkynurenine and commits the aminoacid toward its conversion to QA through the kynurenine pathway (90), which accounts for >90% of tryptophan catabolism (256). Tryptophan is an essential amino acid in protein metabolism, a precursor for the synthesis of the neurotransmitter serotonin and tryptamine, as well as for the synthesis of NAD and the hormone melatonin (257, 258).

**Figure 6:**
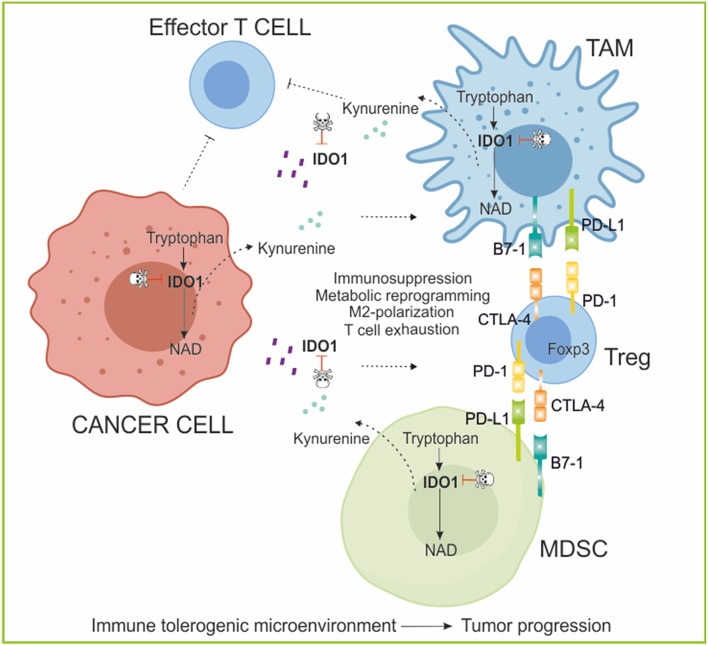
The role of IDO/kynurenine in cancer immunoediting. IDO1 is an enzyme involved in the catabolism of tryptophan (kynurenine pathway). IDO and kynurenine can be secreted by tumor and tolerogenic immune cells in the microenvironment where exert an immunosuppressive function polarizing myeloid cell toward M2 phenotype (TAM; MDSC) and suppressing effector T cell functions, while stimulating expansion and activation of permissive Treg population, increasing immune escape mechanisms (PD-1/PD-L1; CTLA-4/B7-1 crosstalk). Moreover, in the extracellular space, IDO1 depletes the essential amino acid tryptophan from the tumor microenvironment, favoring tumor growth. IDO1 inhibitors in combination with Immunotherapy aim to reverse immunoediting (backward arrow) by inhibiting and activating local immunosuppressive and tumor eradication mechanisms, respectively. PD-1, programmed cell death protein 1; PD-L1, PD-1 ligand; CTLA-4, Cytotoxic T-Lymphocyte Antigen 4; Foxp3, forkhead box P3; TAM, tumor-associated macrophages; MDSC, myeloid-derived suppressive cells.

In recent years, IDO has drawn enormous attention due to its immune regulatory functions (259–261) and summarized in Figure 6. IDO is not constitutively expressed in immune cells. Rather, various stimuli, and signaling pathways induce transcription and translation of metabolically-active IDO enzyme protein. Among them, TLRs, tumor necrosis factor superfamily members (TNFRs), interferon beta receptor (IFNBR), the interferon gamma receptor (IFNGR), transforming growth factor beta receptors (TGFBRs) and the aryl hydrocarbon receptor (AhR) all can activate signaling mechanisms that either induce or maintain IDO expression. NF-KB activation is a central downstream signal of these pathways regulating IDO expression (262).

By catalyzing the initial and rate-limiting step of tryptophan degradation, IDO reduces the local tryptophan concentration and produces immune modulatory tryptophan metabolites (263). In particular, cells expressing IDO and TDO produce the tryptophan catabolite kynurenine that, by interacting with the aryl hydrocarbon receptor expressed by T cells, Tregs and DCs, regulates immunity (264). Additionally, inhibition of CD8^+^ T-cell-mediated cytotoxic function was found to be an important mechanism behind IDO's immune-modulating property (264). Due to the role in regulating T cell response and fate, IDO function is critical in organ and tissue graft survival, in viral infection, in tissue-specific autoimmunity and the promotion of cancer cell survival (265). The biologic function of the IDO pathway was originally described as both counter-regulatory (controlling inflammation) and tolerogenic (creating acquired antigen-specific tolerance in T cells) (257).

Escape from the immune response is essential for cancer progression, however, mechanisms underlying this process remain unclear. Kynurenine in the tumor microenvironment was recently shown to favor immunosuppression (265, 266). Tryptophan catabolism was shown to create an immuno-suppressive milieu in tumors and in tumor-draining lymph nodes through accumulation and secretion of immunosuppressive tryptophan catabolites that bind and activate AhR (267), leading to induction of T-cell anergy, apoptosis, increased conversion of naïve CD4^+^ T cells into Tregs and polarization of DCs and macrophages toward an immunosuppressive phenotype (190, 261, 265, 268).

Clinically, studies of ovarian, lung, colorectal, breast cancer, brain tumors, melanoma, and others have shown that increased expression of IDO was associated with poor survival outcomes (258, 265, 269, 270). In most studies, the ratio of kynurenine to tryptophan was measured in patient plasma as a measure of IDO and TDO activity (156, 267). Moreover, not only tumor can express IDO, but also immune cells including both TAM and MDSC express high levels of IDO, in response to inflammatory cytokines, of which IFN-γ is the most potent inducer, amplifying the circuit of immunosuppression (190, 271, 272).

According to the role of IDO in driving immunosuppression, in the last years IDO became a valid target in cancer therapy (273, 274). Competitive inhibitors of IDO are currently being tested in clinical trials in patients with solid cancer, with the aim of enhancing the efficacy of conventional chemotherapy, vaccine or checkpoint inhibitors (275). Agents currently account for the majority of the trials: indoximod (1-methyl-D-tryptophan), an inhibitor of the IDO pathway (155, 276), epacadostat (INCB024360) (156) and BMS-986205 (158) (Table 1), with encouraging results. Importantly, the use of IDO inhibitors can be also overcome the resistance to immunotherapies targeting immune checkpoints, strongly supporting the combination therapies with IDO inhibitors irrespective of IDO expression by the tumor cells (277). Additional IDO inhibitors are in the development pipeline, as well as agents that may target TDO, or a second isoform of IDO (IDO2) (275).

## Concluding Remarks

Anticancer strategies targeting simultaneously oncogenic and metabolic pathways, de-regulated in cancer cells, seem to be ideal and have shown some promising results. Interestingly, local conditions in the tumor microenvironment affect also metabolic responses of immune cells, favoring immune-tolerance, and immune-escape mechanisms. One of the goals of immunotherapy could be to re-educate the immune system to kill tumors, by reprogramming their metabolism. The network of immunosuppressive mechanisms in the TME is complex, multifactorial, and mutually reinforcing. A better knowledge of the main players of this cross-talk can help in designing more effective combination therapies. In this picture, NAD-metabolizing enzymes are receiving increasing attention to due to their role in conditioning several aspects of immune cell fate and functions. It is foreseeable that modulators/inhibitors of the NADome (summarized in Table 1) will become useful alone or in combination with current anti-cancer therapeutic strategies to regulate both tumor growth and immune populations of TME.

## Author Contributions

All authors listed have made a substantial, direct and intellectual contribution to the work, and approved it for publication.

### Conflict of Interest Statement

The authors declare that the research was conducted in the absence of any commercial or financial relationships that could be construed as a potential conflict of interest.

## References

[1] Hanahan D Coussens LM. Accessories to the crime: functions of cells recruited to the tumor microenvironment. Cancer Cell. (2012) 21:309–22. 10.1016/j.ccr.2012.02.022 22439926

[2] Quail DF Joyce JA. Microenvironmental regulation of tumor progression and metastasis. Nat Med. (2013) 19:1423–37. 10.1038/nm.3394 24202395 PMC3954707

[3] Fridman WH Pages F Sautes-Fridman C Galon J. The immune contexture in human tumours: impact on clinical outcome. Nat Rev Cancer. (2012) 12:298–306. 10.1038/nrc3245 22419253

[4] Nagarsheth N Wicha MS Zou W. Chemokines in the cancer microenvironment and their relevance in cancer immunotherapy. Nat Rev Immunol. (2017) 17:559–72. 10.1038/nri.2017.49 28555670 PMC5731833

[5] Cairns RA Harris IS Mak TW. Regulation of cancer cell metabolism. Nat Rev Cancer. (2011) 11:85–95. 10.1038/nrc2981 21258394

[6] Kareva I Hahnfeldt P The emerging hallmarks of metabolic reprogramming and immune evasion: distinct or linked? Cancer Res. (2013) 73:2737–42. 10.1158/0008-5472.CAN-12-3696 23423980

[7] Boroughs LK Deberardinis RJ. Metabolic pathways promoting cancer cell survival and growth. Nat Cell Biol. (2015) 17:351–9. 10.1038/ncb3124 25774832 PMC4939711

[8] Morandi A Giannoni E Chiarugi P. Nutrient exploitation within the tumor-stroma metabolic crosstalk. Trends Cancer. (2016) 2:736–46. 10.1016/j.trecan.2016.11.001 28741520

[9] Kerkar SP Restifo NP. Cellular constituents of immune escape within the tumor microenvironment. Cancer Res. (2012) 72:3125–30. 10.1158/0008-5472.CAN-11-4094 22721837 PMC6327310

[10] Schiavoni G Gabriele L Mattei F. The tumor microenvironment: a pitch for multiple players. Front Oncol. (2013) 3:90. 10.3389/fonc.2013.00090 23616948 PMC3628362

[11] Armulik A Abramsson A Betsholtz C. Endothelial/pericyte interactions. Circ Res. (2005) 97:512–23. 10.1161/01.RES.0000182903.16652.d7 16166562

[12] Chouaib S Kieda C Benlalam H Noman MZ Mami-Chouaib F Ruegg C. Endothelial cells as key determinants of the tumor microenvironment: interaction with tumor cells, extracellular matrix and immune killer cells. Crit Rev Immunol. (2010) 30:529–45. 10.1615/CritRevImmunol.v30.i6.30 21175416

[13] Cao Z Ding BS Guo P Lee SB Butler JM Casey SC. Angiocrine factors deployed by tumor vascular niche induce B cell lymphoma invasiveness and chemoresistance. Cancer Cell. (2014) 25:350–65. 10.1016/j.ccr.2014.02.005 24651014 PMC4017921

[14] Petrova V Annicchiarico-Petruzzelli M Melino G Amelio I. The hypoxic tumour microenvironment. Oncogenesis. (2018) 7:10. 10.1038/s41389-017-0011-9 29362402 PMC5833859

[15] Hegde PS Wallin JJ Mancao C. Predictive markers of anti-VEGF and emerging role of angiogenesis inhibitors as immunotherapeutics. Semin Cancer Biol. (2018) 52:117–24. 10.1016/j.semcancer.2017.12.002 29229461

[16] Uccelli A Moretta L Pistoia V. Mesenchymal stem cells in health and disease. Nat Rev Immunol. (2008) 8:726–36. 10.1038/nri2395 19172693

[17] Shah K. Mesenchymal stem cells engineered for cancer therapy. Adv Drug Deliv Rev. (2012) 64:739–48. 10.1016/j.addr.2011.06.010 21740940 PMC3395998

[18] Shi Y Du L Lin L Wang Y. Tumour-associated mesenchymal stem/stromal cells: emerging therapeutic targets. Nat Rev Drug Discov. (2017) 16:35–52. 10.1038/nrd.2016.193 27811929

[19] Quante M Tu SP Tomita H Gonda T Wang SS Takashi S. Bone marrow-derived myofibroblasts contribute to the mesenchymal stem cell niche and promote tumor growth. Cancer Cell. (2011) 19:257–72. 10.1016/j.ccr.2011.01.020 21316604 PMC3060401

[20] Su S Chen J Yao H Liu J Yu S Lao L. CD10(+)GPR77(+) Cancer-associated fibroblasts promote cancer formation and chemoresistance by sustaining cancer stemness. Cell. (2018) 172:841–56.e816. 10.1016/j.cell.2018.01.009 29395328

[21] Chen X Song E. Turning foes to friends: targeting cancer-associated fibroblasts. Nat Rev Drug Discov. (2019) 18:99–115. 10.1038/s41573-018-0004-1 30470818

[22] Giannoni E Bianchini F Masieri L Serni S Torre E Calorini L. Reciprocal activation of prostate cancer cells and cancer-associated fibroblasts stimulates epithelial-mesenchymal transition and cancer stemness. Cancer Res. (2010) 70:6945–56. 10.1158/0008-5472.CAN-10-0785 20699369

[23] Ohlund D Elyada E Tuveson D. Fibroblast heterogeneity in the cancer wound. J Exp Med. (2014) 211:1503–23. 10.1084/jem.20140692 25071162 PMC4113948

[24] Molodtsov A Turk MJ. Tissue resident CD8 memory T cell responses in cancer and autoimmunity. Front Immunol. (2018) 9:2810. 10.3389/fimmu.2018.02810 30555481 PMC6281983

[25] Gentles AJ Newman AM Liu CL Bratman SV Feng W Kim D. The prognostic landscape of genes and infiltrating immune cells across human cancers. Nat Med. (2015) 21:938–45. 10.1038/nm.3909 26193342 PMC4852857

[26] Speiser DE Ho PC Verdeil G. Regulatory circuits of T cell function in cancer. Nat Rev Immunol. (2016) 16:599–611. 10.1038/nri.2016.80 27526640

[27] Zhao S Xu W Jiang W Yu W Lin Y Zhang T. Regulation of cellular metabolism by protein lysine acetylation. Science. (2010) 327:1000–4. 10.1126/science.1179689 20167786 PMC3232675

[28] Gooden MJ De Bock GH Leffers N Daemen T Nijman HW. The prognostic influence of tumour-infiltrating lymphocytes in cancer: a systematic review with meta-analysis. Br J Cancer. (2011) 105:93–103. 10.1038/bjc.2011.189 21629244 PMC3137407

[29] Klebanoff CA Gattinoni L Torabi-Parizi P Kerstann K Cardones AR Finkelstein SE. Central memory self/tumor-reactive CD8+ T cells confer superior antitumor immunity compared with effector memory T cells. Proc Natl Acad Sci USA. (2005) 102:9571–6. 10.1073/pnas.0503726102 15980149 PMC1172264

[30] Whiteside TL. Disarming suppressor cells to improve immunotherapy. Cancer Immunol Immunother. (2012) 61:283–8. 10.1007/s00262-011-1171-7 22146892 PMC11028463

[31] Wherry EJ Kurachi M. Molecular and cellular insights into T cell exhaustion. Nat Rev Immunol. (2015) 15:486–99. 10.1038/nri3862 26205583 PMC4889009

[32] Sakaguchi S Yamaguchi T Nomura T Ono M. Regulatory T cells and immune tolerance. Cell. (2008) 133:775–87. 10.1016/j.cell.2008.05.009 18510923

[33] Hsieh CS Lee HM Lio CW. Selection of regulatory T cells in the thymus. Nat Rev Immunol. (2012) 12:157–67. 10.1038/nri3155 22322317

[34] Newton R Priyadharshini B Turka LA. Immunometabolism of regulatory T cells. Nat Immunol. (2016) 17:618–25. 10.1038/ni.3466 27196520 PMC5006394

[35] Shang B Liu Y Jiang SJ Liu Y. Prognostic value of tumor-infiltrating FoxP3+ regulatory T cells in cancers: a systematic review and meta-analysis. Sci Rep. (2015) 5:15179. 10.1038/srep15179 26462617 PMC4604472

[36] Huang L Xu H Peng G. TLR-mediated metabolic reprogramming in the tumor microenvironment: potential novel strategies for cancer immunotherapy. Cell Mol Immunol. (2018) 15:428–37. 10.1038/cmi.2018.4 29553135 PMC6068099

[37] Von Boehmer H Daniel C. Therapeutic opportunities for manipulating T(Reg) cells in autoimmunity and cancer. Nat Rev Drug Discov. (2013) 12:51–63. 10.1038/nrd3683 23274471

[38] Sica A Larghi P Mancino A Rubino L Porta C Totaro MG. Macrophage polarization in tumour progression. Semin Cancer Biol. (2008) 18:349–55. 10.1016/j.semcancer.2008.03.004 18467122

[39] Qian BZ Pollard JW. Macrophage diversity enhances tumor progression and metastasis. Cell. (2010) 141:39–51. 10.1016/j.cell.2010.03.014 20371344 PMC4994190

[40] Biswas SK Mantovani A. Macrophage plasticity and interaction with lymphocyte subsets: cancer as a paradigm. Nat Immunol. (2010) 11:889–96. 10.1038/ni.1937 20856220

[41] Escribese MM Casas M Corbi AL. Influence of low oxygen tensions on macrophage polarization. Immunobiology. (2012) 217:1233–40. 10.1016/j.imbio.2012.07.002 22889513

[42] Comito G Giannoni E Segura CP Barcellos-De-Souza P Raspollini MR Baroni G. Cancer-associated fibroblasts and M2-polarized macrophages synergize during prostate carcinoma progression. Oncogene. (2014) 33:2423–31. 10.1038/onc.2013.191 23728338

[43] Condeelis J Pollard JW. Macrophages: obligate partners for tumor cell migration, invasion, and metastasis. Cell. (2006) 124:263–6. 10.1016/j.cell.2006.01.007 16439202

[44] Goswami S Sahai E Wyckoff JB Cammer M Cox D Pixley FJ. Macrophages promote the invasion of breast carcinoma cells via a colony-stimulating factor-1/epidermal growth factor paracrine loop. Cancer Res. (2005) 65:5278–83. 10.1158/0008-5472.CAN-04-1853 15958574

[45] Cassetta L Pollard JW. Targeting macrophages: therapeutic approaches in cancer. Nat Rev Drug Discov. (2018) 17:887–904. 10.1038/nrd.2018.169 30361552

[46] Motz GT Coukos G. Deciphering and reversing tumor immune suppression. Immunity. (2013) 39:61–73. 10.1016/j.immuni.2013.07.005 23890064 PMC3782392

[47] Talmadge JE Gabrilovich DI. History of myeloid-derived suppressor cells. Nat Rev Cancer. (2013) 13:739–52. 10.1038/nrc3581 24060865 PMC4358792

[48] Gabrilovich DI Ostrand-Rosenberg S Bronte V. Coordinated regulation of myeloid cells by tumours. Nat Rev Immunol. (2012) 12:253–68. 10.1038/nri3175 22437938 PMC3587148

[49] Foudi A Jarrier P Zhang Y Wittner M Geay JF Lecluse Y. Reduced retention of radioprotective hematopoietic cells within the bone marrow microenvironment in CXCR4-/- chimeric mice. Blood. (2006) 107:2243–51. 10.1182/blood-2005-02-0581 16291599

[50] Safari E Ghorghanlu S Ahmadi-Khiavi H Mehranfar S Rezaei R Motallebnezhad M. Myeloid-derived suppressor cells and tumor: current knowledge and future perspectives. J Cell Physiol. (2018) 234:9966–81. 10.1002/jcp.27923 30537008

[51] Wang Y Ding Y Guo N Wang S. MDSCs: key criminals of tumor pre-metastatic niche formation. Front Immunol. (2019) 10:172. 10.3389/fimmu.2019.00172 30792719 PMC6374299

[52] Peled A Abraham M Avivi I Rowe JM Beider K Wald H. The high-affinity CXCR4 antagonist BKT140 is safe and induces a robust mobilization of human CD34+ cells in patients with multiple myeloma. Clin Cancer Res. (2014) 20:469–79. 10.1158/1078-0432.CCR-13-1302 24246358

[53] Serafini P Borrello I Bronte V. Myeloid suppressor cells in cancer: recruitment, phenotype, properties, and mechanisms of immune suppression. Semin Cancer Biol. (2006) 16:53–65. 10.1016/j.semcancer.2005.07.005 16168663

[54] Fridlender ZG Sun J Kim S Kapoor V Cheng G Ling L. Polarization of tumor-associated neutrophil phenotype by TGF-beta: N1 versus N2 TAN. Cancer Cell. (2009) 16:183–94. 10.1016/j.ccr.2009.06.017 19732719 PMC2754404

[55] Gregory AD Houghton AM. Tumor-associated neutrophils: new targets for cancer therapy. Cancer Res. (2011) 71:2411–6. 10.1158/0008-5472.CAN-10-2583 21427354

[56] Hurt B Schulick R Edil B El Kasmi KC Barnett C Jr. Cancer-promoting mechanisms of tumor-associated neutrophils. Am J Surg. (2017) 214:938–44. 10.1016/j.amjsurg.2017.08.003 28830617

[57] Semenza GL. HIF-1 mediates metabolic responses to intratumoral hypoxia and oncogenic mutations. J Clin Invest. (2013) 123:3664–71. 10.1172/JCI67230 23999440 PMC3754249

[58] Sormendi S Wielockx B. Hypoxia pathway proteins as central mediators of metabolism in the tumor cells and their microenvironment. Front Immunol. (2018) 9:40. 10.3389/fimmu.2018.00040 29434587 PMC5796897

[59] Pavlova NN Thompson CB. The emerging hallmarks of cancer metabolism. Cell Metab. (2016) 23:27–47. 10.1016/j.cmet.2015.12.006 26771115 PMC4715268

[60] Gottfried E Kunz-Schughart LA Ebner S Mueller-Klieser W Hoves S Andreesen R. Tumor-derived lactic acid modulates dendritic cell activation and antigen expression. Blood. (2006) 107:2013–21. 10.1182/blood-2005-05-1795 16278308

[61] Fischer K Hoffmann P Voelkl S Meidenbauer N Ammer J Edinger M. Inhibitory effect of tumor cell-derived lactic acid on human T cells. Blood. (2007) 109:3812–9. 10.1182/blood-2006-07-035972 17255361

[62] Goetze K Walenta S Ksiazkiewicz M Kunz-Schughart LA Mueller-Klieser W. Lactate enhances motility of tumor cells and inhibits monocyte migration and cytokine release. Int J Oncol. (2011) 39:453–63. 10.3892/ijo.2011.1055 21617859

[63] Colegio OR Chu NQ Szabo AL Chu T Rhebergen AM Jairam V. Functional polarization of tumour-associated macrophages by tumour-derived lactic acid. Nature. (2014) 513:559–63. 10.1038/nature13490 25043024 PMC4301845

[64] Majmundar AJ Wong WJ Simon MC. Hypoxia-inducible factors and the response to hypoxic stress. Mol Cell. (2010) 40:294–309. 10.1016/j.molcel.2010.09.022 20965423 PMC3143508

[65] Belaiba RS Bonello S Zahringer C Schmidt S Hess J Kietzmann T. Hypoxia up-regulates hypoxia-inducible factor-1alpha transcription by involving phosphatidylinositol 3-kinase and nuclear factor kappaB in pulmonary artery smooth muscle cells. Mol Biol Cell. (2007) 18:4691–7. 10.1091/mbc.e07-04-0391 17898080 PMC2096613

[66] Porporato PE Payen VL De Saedeleer CJ Preat V Thissen JP Feron O. Lactate stimulates angiogenesis and accelerates the healing of superficial and ischemic wounds in mice. Angiogenesis. (2012) 15:581–92. 10.1007/s10456-012-9282-0 22660894

[67] Kato Y Ozawa S Miyamoto C Maehata Y Suzuki A Maeda T. Acidic extracellular microenvironment and cancer. Cancer Cell Int. (2013) 13:89. 10.1186/1475-2867-13-89 24004445 PMC3849184

[68] Swietach P Hulikova A Vaughan-Jones RD Harris AL. New insights into the physiological role of carbonic anhydrase IX in tumour pH regulation. Oncogene. (2010) 29:6509–21. 10.1038/onc.2010.455 20890298

[69] Chiche J Brahimi-Horn MC Pouyssegur J. Tumour hypoxia induces a metabolic shift causing acidosis: a common feature in cancer. J Cell Mol Med. (2010) 14:771–94. 10.1111/j.1582-4934.2009.00994.x 20015196 PMC3823111

[70] Pavlides S Whitaker-Menezes D Castello-Cros R Flomenberg N Witkiewicz AK Frank PG. The reverse Warburg effect: aerobic glycolysis in cancer associated fibroblasts and the tumor stroma. Cell Cycle. (2009) 8:3984–4001. 10.4161/cc.8.23.10238 19923890

[71] Gladden LB. Lactate metabolism: a new paradigm for the third millennium. J Physiol. (2004) 558:5–30. 10.1113/jphysiol.2003.058701 15131240 PMC1664920

[72] Jia D Park JH Jung KH Levine H Kaipparettu BA. Elucidating the metabolic plasticity of cancer: mitochondrial reprogramming and hybrid metabolic states. Cells. (2018) 7:21. 10.3390/cells7030021 29534029 PMC5870353

[73] Fu Y Liu S Yin S Niu W Xiong W Tan M. The reverse Warburg effect is likely to be an Achilles' heel of cancer that can be exploited for cancer therapy. Oncotarget. (2017) 8:57813–25. 10.18632/oncotarget.18175 28915713 PMC5593685

[74] Fiaschi T Marini A Giannoni E Taddei ML Gandellini P De Donatis A. Reciprocal metabolic reprogramming through lactate shuttle coordinately influences tumor-stroma interplay. Cancer Res. (2012) 72:5130–40. 10.1158/0008-5472.CAN-12-1949 22850421

[75] Suh DH Kim HS Kim B Song YS. Metabolic orchestration between cancer cells and tumor microenvironment as a co-evolutionary source of chemoresistance in ovarian cancer: a therapeutic implication. Biochem Pharmacol. (2014) 92:43–54. 10.1016/j.bcp.2014.08.011 25168677

[76] Penkert J Ripperger T Schieck M Schlegelberger B Steinemann D Illig T. On metabolic reprogramming and tumor biology: a comprehensive survey of metabolism in breast cancer. Oncotarget. (2016) 7:67626–49. 10.18632/oncotarget.11759 27590516 PMC5341901

[77] Phan AT Goldrath AW Glass CK. Metabolic and epigenetic coordination of T cell and macrophage Immunity. (2017) 46:714–29. 10.1016/j.immuni.2017.04.016 28514673 PMC5505665

[78] Ho PC Bihuniak JD Macintyre AN Staron M Liu X Amezquita R. Phosphoenolpyruvate is a metabolic checkpoint of anti-tumor T cell responses. Cell. (2015) 162:1217–28. 10.1016/j.cell.2015.08.012 26321681 PMC4567953

[79] Scharping NE Menk AV Moreci RS Whetstone RD Dadey RE Watkins SC The tumor microenvironment represses T cell mitochondrial biogenesis to drive intratumoral T cell metabolic insufficiency and dysfunction. Immunity. (2016) 45:701–3. 10.1016/j.immuni.2016.08.009 27653602

[80] Beckermann KE Dudzinski SO Rathmell JC. Dysfunctional T cell metabolism in the tumor microenvironment. Cytokine Growth Factor Rev. (2017) 35:7–14. 10.1016/j.cytogfr.2017.04.003 28456467 PMC5710836

[81] Di Virgilio F Sarti AC Falzoni S De Marchi E Adinolfi E. Extracellular ATP and P2 purinergic signalling in the tumour microenvironment. Nat Rev Cancer. (2018) 18:601–18. 10.1038/s41568-018-0037-0 30006588

[82] Deaglio S Dwyer KM Gao W Friedman D Usheva A Erat A. Adenosine generation catalyzed by CD39 and CD73 expressed on regulatory T cells mediates immune suppression. J Exp Med. (2007) 204:1257–65. 10.1084/jem.20062512 17502665 PMC2118603

[83] Vijayan D Young A Teng MWL Smyth MJ Targeting immunosuppressive adenosine in cancer. Nat Rev Cancer. (2017) 17:765 10.1038/nrc.2017.86 29162946

[84] Serra S Vaisitti T Audrito V Bologna C Buonincontri R Chen SS. Adenosine signaling mediates hypoxic responses in the chronic lymphocytic leukemia microenvironment. Blood Adv. (2016) 1:47–61. 10.1182/bloodadvances.2016000984 29296695 PMC5744057

[85] Vaupel P Multhoff G. Accomplices of the hypoxic tumor microenvironment compromising antitumor immunity: adenosine, lactate, acidosis, vascular endothelial growth factor, potassium ions, and phosphatidylserine. Front Immunol. (2017) 8:1887. 10.3389/fimmu.2017.01887 29312351 PMC5742577

[86] Gossmann TI Ziegler M Puntervoll P De Figueiredo LF Schuster S Heiland I. NAD(+) biosynthesis and salvage–a phylogenetic perspective. Febs J. (2012) 279:3355–63. 10.1111/j.1742-4658.2012.08559.x 22404877

[87] Sharif T Martell E Dai C Ghassemi-Rad MS Kennedy BE Lee PWK. Regulation of cancer and cancer-related genes via NAD(). Antioxid Redox Signal. (2018) 30:906–23. 10.1089/ars.2017.7478 29334761

[88] Canto C Menzies KJ Auwerx J. NAD(+) Metabolism and the control of energy homeostasis: a balancing act between mitochondria and the nucleus. Cell Metab. (2015) 22:31–53. 10.1016/j.cmet.2015.05.023 26118927 PMC4487780

[89] Nikiforov A Kulikova V Ziegler M. The human NAD metabolome: functions, metabolism and compartmentalization. Crit Rev Biochem Mol Biol. (2015) 50:284–97. 10.3109/10409238.2015.1028612 25837229 PMC4673589

[90] Ruggieri S Orsomando G Sorci L Raffaelli N. Regulation of NAD biosynthetic enzymes modulates NAD-sensing processes to shape mammalian cell physiology under varying biological cues. Biochim Biophys Acta. (2015) 1854:1138–49. 10.1016/j.bbapap.2015.02.021 25770681

[91] Magni G Orsomando G Raffelli N Ruggieri S. Enzymology of mammalian NAD metabolism in health and disease. Front Biosci. (2008) 13:6135–54. 10.2741/3143 18508649

[92] Houtkooper RH Canto C Wanders RJ Auwerx J. The secret life of NAD+: an old metabolite controlling new metabolic signaling pathways. Endocr Rev. (2010) 31:194–223. 10.1210/er.2009-0026 20007326 PMC2852209

[93] Chiarugi A Dolle C Felici R Ziegler M. The NAD metabolome–a key determinant of cancer cell biology. Nat Rev Cancer. (2012) 12:741–52. 10.1038/nrc3340 23018234

[94] Haag F Adriouch S Brass A Jung C Moller S Scheuplein F. Extracellular NAD and ATP: partners in immune cell modulation. Purinergic Signal. (2007) 3:71–81. 10.1007/s11302-006-9038-7 18404420 PMC2096762

[95] Grahnert A Klein C Schilling E Wehrhahn J Hauschildt S. Review: NAD +: a modulator of immune functions. Innate Immun. (2011) 17:212–33. 10.1177/1753425910361989 20388721

[96] Burgos ES Schramm VL. Weak coupling of ATP hydrolysis to the chemical equilibrium of human nicotinamide phosphoribosyltransferase. Biochemistry. (2008) 47:11086–96. 10.1021/bi801198m 18823127 PMC2657875

[97] Kitani T Okuno S Fujisawa H. Growth phase-dependent changes in the subcellular localization of pre-B-cell colony-enhancing factor. FEBS Lett. (2003) 544:74–8. 10.1016/S0014-5793(03)00476-9 12782293

[98] Revollo JR Korner A Mills KF Satoh A Wang T Garten A. Nampt/PBEF/Visfatin regulates insulin secretion in beta cells as a systemic NAD biosynthetic enzyme. Cell Metab. (2007) 6:363–75. 10.1016/j.cmet.2007.09.003 17983582 PMC2098698

[99] Yang H Yang T Baur JA Perez E Matsui T Carmona JJ. Nutrient-sensitive mitochondrial NAD+ levels dictate cell survival. Cell. (2007) 130:1095–107. 10.1016/j.cell.2007.07.035 17889652 PMC3366687

[100] Pittelli M Formentini L Faraco G Lapucci A Rapizzi E Cialdai F. Inhibition of nicotinamide phosphoribosyltransferase: cellular bioenergetics reveals a mitochondrial insensitive NAD pool. J Biol Chem. (2010) 285:34106–14. 10.1074/jbc.M110.136739 20724478 PMC2962509

[101] Davila A Liu L Chellappa K Redpath P Nakamaru-Ogiso E Paolella LM. Nicotinamide adenine dinucleotide is transported into mammalian mitochondria. Elife. (2018) 7. 10.7554/eLife.33246 29893687 PMC6013257

[102] Kennedy BE Sharif T Martell E Dai C Kim Y Lee PW. NAD(+) salvage pathway in cancer metabolism and therapy. Pharmacol Res. (2016) 114:274–83. 10.1016/j.phrs.2016.10.027 27816507

[103] Garten A Schuster S Penke M Gorski T De Giorgis T Kiess W. Physiological and pathophysiological roles of NAMPT and NAD metabolism. Nat Rev Endocrinol. (2015) 11:535–46. 10.1038/nrendo.2015.117 26215259

[104] Tanaka M Nozaki M Fukuhara A Segawa K Aoki N Matsuda M. Visfatin is released from 3T3-L1 adipocytes via a non-classical pathway. Biochem Biophys Res Commun. (2007) 359:194–201. 10.1016/j.bbrc.2007.05.096 17543285

[105] Camp SM Ceco E Evenoski CL Danilov SM Zhou T Chiang ET. Unique toll-like receptor 4 activation by NAMPT/PBEF induces NFkappaB signaling and inflammatory lung injury. Sci Rep. (2015) 5:13135. 10.1038/srep13135 26272519 PMC4536637

[106] Hara N Yamada K Shibata T Osago H Tsuchiya M Nicotinamide phosphoribosyltransferase/visfatin does not catalyze nicotinamide mononucleotide formation in blood plasma. PLoS ONE. (2011) 6:e22781 10.1371/journal.pone.0022781 21826208 PMC3149623

[107] Samal B Sun Y Stearns G Xie C Suggs S Mcniece I. Cloning and characterization of the cDNA encoding a novel human pre-B-cell colony-enhancing factor. Mol Cell Biol. (1994) 14:1431–7. 10.1128/MCB.14.2.1431 8289818 PMC358498

[108] Fukuhara A Matsuda M Nishizawa M Segawa K Tanaka M Kishimoto K. Visfatin: a protein secreted by visceral fat that mimics the effects of insulin. Science. (2005) 307:426–30. 10.1126/science.1097243 15604363

[109] Sun Z Lei H Zhang Z. Pre-B cell colony enhancing factor. (PBEF), a cytokine with multiple physiological functions. Cytokine Growth Factor Rev. (2013) 24:433–42. 10.1016/j.cytogfr.2013.05.006 23787158 PMC3791181

[110] Ognjanovic S Bao S Yamamoto SY Garibay-Tupas J Samal B Bryant-Greenwood GD. Genomic organization of the gene coding for human pre-B-cell colony enhancing factor and expression in human fetal membranes. J Mol Endocrinol. (2001) 26:107–17. 10.1677/jme.0.0260107 11241162

[111] Iqbal J Zaidi M. TNF regulates cellular NAD+ metabolism in primary macrophages. Biochem Biophys Res Commun. (2006) 342:1312–8. 10.1016/j.bbrc.2006.02.109 16516847

[112] Nowell MA Richards PJ Fielding CA Ognjanovic S Topley N Williams AS. Regulation of pre-B cell colony-enhancing factor by STAT-3-dependent interleukin-6 trans-signaling: implications in the pathogenesis of rheumatoid arthritis. Arthritis Rheum. (2006) 54:2084–95. 10.1002/art.21942 16802343

[113] Gosset M Berenbaum F Salvat C Sautet A Pigenet A Tahiri K. Crucial role of visfatin/pre-B cell colony-enhancing factor in matrix degradation and prostaglandin E2 synthesis in chondrocytes: possible influence on osteoarthritis. Arthritis Rheum. (2008) 58:1399–409. 10.1002/art.23431 18438860

[114] Moschen AR Kaser A Enrich B Mosheimer B Theurl M Niederegger H. Visfatin, an adipocytokine with proinflammatory and immunomodulating properties. J Immunol. (2007) 178:1748–58. 10.4049/jimmunol.178.3.1748 17237424

[115] Fan Y Meng S Wang Y Cao J Wang C. Visfatin/PBEF/Nampt induces EMMPRIN and MMP-9 production in macrophages via the NAMPT-MAPK. (p38, ERK1/2)-NF-kappaB signaling pathway. Int J Mol Med. (2011) 27:607–15. 10.3892/ijmm.2011.621 21327328

[116] Murray PJ Allen JE Biswas SK Fisher EA Gilroy DW Goerdt S. Macrophage activation and polarization: nomenclature and experimental guidelines. Immunity. (2014) 41:14–20. 10.1016/j.immuni.2014.06.008 25035950 PMC4123412

[117] Diskin C Palsson-Mcdermott EM. Metabolic modulation in macrophage effector function. Front Immunol. (2018) 9:270. 10.3389/fimmu.2018.00270 29520272 PMC5827535

[118] Stunault MI Bories G Guinamard RR Ivanov S. Metabolism plays a key role during macrophage activation. Mediators Inflamm. (2018) 2018:2426138. 10.1155/2018/2426138 30647530 PMC6311794

[119] Suzuki H Hisamatsu T Chiba S Mori K Kitazume MT Shimamura K. Glycolytic pathway affects differentiation of human monocytes to regulatory macrophages. Immunol Lett. (2016) 176:18–27. 10.1016/j.imlet.2016.05.009 27208804

[120] Kelly B O'neill LA. Metabolic reprogramming in macrophages and dendritic cells in innate immunity. Cell Res. (2015) 25:771–84. 10.1038/cr.2015.68 26045163 PMC4493277

[121] Van Den Bossche J Baardman J De Winther MP Metabolic characterization of polarized M1 and M2 bone marrow-derived macrophages using real-time extracellular flux analysis. J Vis Exp. (2015) 53424. 10.3791/53424 PMC4692751 26649578

[122] Hamers AJ Pillai AB. A sweet alternative: maintaining M2 macrophage polarization. Sci Immunol. (2018) 3:eaav7759. 10.1126/sciimmunol.aav7759 30389801 PMC7861128

[123] Jha AK Huang SC Sergushichev A Lampropoulou V Ivanova Y Loginicheva E. Network integration of parallel metabolic and transcriptional data reveals metabolic modules that regulate macrophage polarization. Immunity. (2015) 42:419–30. 10.1016/j.immuni.2015.02.005 25786174

[124] Xu Q Choksi S Qu J Jang J Choe M Banfi B. NADPH oxidases are essential for macrophage differentiation. J Biol Chem. (2016) 291:20030–41. 10.1074/jbc.M116.731216 27489105 PMC5025689

[125] Huang LE Arany Z Livingston DM Bunn HF. Activation of hypoxia-inducible transcription factor depends primarily upon redox-sensitive stabilization of its alpha subunit. J Biol Chem. (1996) 271:32253–9. 10.1074/jbc.271.50.32253 8943284

[126] Kim JW Tchernyshyov I Semenza GL Dang CV. HIF-1-mediated expression of pyruvate dehydrogenase kinase: a metabolic switch required for cellular adaptation to hypoxia. Cell Metab. (2006) 3:177–85. 10.1016/j.cmet.2006.02.002 16517405

[127] Rius J Guma M Schachtrup C Akassoglou K Zinkernagel AS Nizet V. NF-kappaB links innate immunity to the hypoxic response through transcriptional regulation of HIF-1alpha. Nature. (2008) 453:807–11. 10.1038/nature06905 18432192 PMC2669289

[128] Vats D Mukundan L Odegaard JI Zhang L Smith KL Morel CR. Oxidative metabolism and PGC-1beta attenuate macrophage-mediated inflammation. Cell Metab. (2006) 4:13–24. 10.1016/j.cmet.2006.05.011 16814729 PMC1904486

[129] Skokowa J Lan D Thakur BK Wang F Gupta K Cario G. NAMPT is essential for the G-CSF-induced myeloid differentiation via a NAD(+)-sirtuin-1-dependent pathway. Nat Med. (2009) 15:151–8. 10.1038/nm.1913 19182797

[130] Audrito V Serra S Brusa D Mazzola F Arruga F Vaisitti T. Extracellular nicotinamide phosphoribosyltransferase. (NAMPT) promotes M2 macrophage polarization in chronic lymphocytic leukemia. Blood. (2015) 125:111–23. 10.1182/blood-2014-07-589069 25368373

[131] Travelli C Colombo G Mola S Genazzani AA Porta C NAMPT: a pleiotropic modulator of monocytes and macrophages. Pharmacol Res. (2018) 135:25–36. 10.1016/j.phrs.2018.06.022 30031171

[132] Travelli C Consonni FM Sangaletti S Storto M Morlacchi S Grolla AA. Nicotinamide phosphoribosyltransferase. (NAMPT) acts as a metabolic gate for mobilization of myeloid-derived suppressor cells. Cancer Res. (2019) 79:1938–51 10.1158/0008-5472.CAN-18-1544 30777853

[133] Fernandes CA Fievez L Neyrinck AM Delzenne NM Bureau F Vanbever R. Sirtuin inhibition attenuates the production of inflammatory cytokines in lipopolysaccharide-stimulated macrophages. Biochem Biophys Res Commun. (2012) 420:857–61. 10.1016/j.bbrc.2012.03.088 22469470

[134] Liu TF Vachharajani VT Yoza BK Mccall CE. NAD+-dependent sirtuin 1 and 6 proteins coordinate a switch from glucose to fatty acid oxidation during the acute inflammatory response. J Biol Chem. (2012) 287:25758–69. 10.1074/jbc.M112.362343 22700961 PMC3406663

[135] Weidemann A Johnson RS. Biology of HIF-1alpha. Cell Death Differ. (2008) 15:621–7. 10.1038/cdd.2008.12 18259201

[136] Gleyzer N Scarpulla RC. PGC-1-related coactivator. (PRC), a sensor of metabolic stress, orchestrates a redox-sensitive program of inflammatory gene expression. J Biol Chem. (2011) 286:39715–25. 10.1074/jbc.M111.291575 21937425 PMC3220587

[137] Li Y Zhang Y Dorweiler B Cui D Wang T Woo CW. Extracellular Nampt promotes macrophage survival via a nonenzymatic interleukin-6/STAT3 signaling mechanism. J Biol Chem. (2008) 283:34833–43. 10.1074/jbc.M805866200 18945671 PMC2596403

[138] Koch C Samareh B Morishima T Mir P Kanz L Zeidler C GM-CSF treatment is not effective in congenital neutropenia patients due to its inability to activate NAMPT signaling. Ann Hematol. (2017) 96:345–53. 10.1007/s00277-016-2894-5 27966038

[139] Galli U Travelli C Massarotti A Fakhfouri G Rahimian R Tron GC. Medicinal chemistry of nicotinamide phosphoribosyltransferase. (NAMPT) inhibitors. J Med Chem. (2013) 56:6279–96. 10.1021/jm4001049 23679915

[140] Watson M Roulston A Belec L Billot X Marcellus R Bedard D. The small molecule GMX1778 is a potent inhibitor of NAD+ biosynthesis: strategy for enhanced therapy in nicotinic acid phosphoribosyltransferase 1-deficient tumors. Mol Cell Biol. (2009) 29:5872–88. 10.1128/MCB.00112-09 19703994 PMC2772749

[141] Dalamaga M Christodoulatos GS Mantzoros CS. The role of extracellular and intracellular Nicotinamide phosphoribosyl-transferase in cancer: diagnostic and therapeutic perspectives and challenges. Metabolism. (2018) 82:72–87. 10.1016/j.metabol.2018.01.001 29330025

[142] Abu Aboud O Chen CH Senapedis W Baloglu E Argueta C Weiss RH Dual and specific inhibition of NAMPT and PAK4 By KPT-9274 decreases kidney cancer growth. Mol Cancer Ther. (2016) 15:2119–29. 10.1158/1535-7163.MCT-16-0197 27390344 PMC5010932

[143] Gibson AE Mendoza A Korotchkina L Chernova O Heste CM Targeting nicotinamide phosphoribosyltransferase (NAMPT) with OT-82 in Ewing sarcoma [abstract]. In: Proceedings of the American Association for Cancer Research Annual Meeting 2018; 2018 Apr 14-18. Chicago, IL; Philadelphia (PA): AACR Cancer Res. (2018) 78(13 Suppl.):5477 10.1158/1538-7445.AM2018-5477

[144] Pillai VB Sundaresan NR Kim G Samant S Moreno-Vinasco L Garcia JG. Nampt secreted from cardiomyocytes promotes development of cardiac hypertrophy and adverse ventricular remodeling. Am J Physiol Heart Circ Physiol. (2013) 304:H415–26. 10.1152/ajpheart.00468.2012 23203961 PMC3774498

[145] Krejcik J Casneuf T Nijhof IS Verbist B Bald J Plesner T. Daratumumab depletes CD38+ immune regulatory cells, promotes T-cell expansion, and skews T-cell repertoire in multiple myeloma. Blood. (2016) 128:384–94. 10.1182/blood-2015-12-687749 27222480 PMC4957162

[146] Martin T Baz R Benson DM Lendvai N Wolf J Munster P. A phase 1b study of isatuximab plus lenalidomide and dexamethasone for relapsed/refractory multiple myeloma. Blood. (2017) 129:3294–303. 10.1182/blood-2016-09-740787 28483761 PMC5482100

[147] Boxhammer R Weirather J Steidl S Endell J MOR202, a human anti-CD38 monoclonal antibody, mediates potent tumoricidal activity in vivo and shows synergistic efficacy in combination with different antineoplastic compounds. Blood. (2015) 126:3015.

[148] Escande C Nin V Price NL Capellini V Gomes AP Barbosa MT. Flavonoid apigenin is an inhibitor of the NAD^+^ase CD38: implications for cellular NAD+ metabolism, protein acetylation, and treatment of metabolic syndrome. Diabetes. (2013) 62:1084–93. 10.2337/db12-1139 23172919 PMC3609577

[149] Heltweg B Gatbonton T Schuler AD Posakony J Li H Goehle S. Antitumor activity of a small-molecule inhibitor of human silent information regulator 2 enzymes. Cancer Res. (2006) 66:4368–77. 10.1158/0008-5472.CAN-05-3617 16618762

[150] Wang J Kim TH Ahn MY Lee J Jung JH Choi WS. Sirtinol, a class III HDAC inhibitor, induces apoptotic and autophagic cell death in MCF-7 human breast cancer cells. Int J Oncol. (2012) 41:1101–9. 10.3892/ijo.2012.1534 22751989

[151] Lara E Mai A Calvanese V Altucci L Lopez-Nieva P Martinez-Chantar ML. Salermide, a Sirtuin inhibitor with a strong cancer-specific proapoptotic effect. Oncogene. (2009) 28:781–91. 10.1038/onc.2008.436 19060927

[152] Lain S Hollick JJ Campbell J Staples OD Higgins M Aoubala M. Discovery, in vivo activity, and mechanism of action of a small-molecule p53 activator. Cancer Cell. (2008) 13:454–63. 10.1016/j.ccr.2008.03.004 18455128 PMC2742717

[153] Peck B Chen CY Ho KK Di Fruscia P Myatt SS Coombes RC. SIRT inhibitors induce cell death and p53 acetylation through targeting both SIRT1 and SIRT2. Mol Cancer Ther. (2010) 9:844–55. 10.1158/1535-7163.MCT-09-0971 20371709

[154] Avalos JL Bever KM Wolberger C. Mechanism of sirtuin inhibition by nicotinamide: altering the NAD^(+)^ cosubstrate specificity of a Sir2 enzyme. Mol Cell. (2005) 17:855–68. 10.1016/j.molcel.2005.02.022 15780941

[155] Wainwright DA Chang AL Dey M Balyasnikova IV Kim CK Tobias A. Durable therapeutic efficacy utilizing combinatorial blockade against IDO, CTLA-4, and PD-L1 in mice with brain tumors. Clin Cancer Res. (2014) 20:5290–301. 10.1158/1078-0432.CCR-14-0514 24691018 PMC4182350

[156] Liu X Shin N Koblish HK Yang G Wang Q Wang K. Selective inhibition of IDO1 effectively regulates mediators of antitumor immunity. Blood. (2010) 115:3520–30. 10.1182/blood-2009-09-246124 20197554

[157] Prendergast GC Malachowski WJ Mondal A Scherle P Muller AJ. Indoleamine 2,3-Dioxygenase and its therapeutic inhibition in cancer. Int Rev Cell Mol Biol. (2018) 336:175–203. 10.1016/bs.ircmb.2017.07.004 29413890 PMC6054468

[158] Tabernero J Luke JJ Joshua AM Varga AI Moreno V Desai J BMS-986205, an indoleamine 2,3-dioxygenase 1 inhibitor (IDO1i), in combination with nivolumab (NIVO): Updated safety across all tumor cohorts and efficacy in pts with advanced bladder cancer (advBC). J Clin Oncol. (2018) 36(15_suppl):4512 10.1200/JCO.2018.36.15_suppl.4512

[159] Hasmann M Schemainda I. FK866, a highly specific noncompetitive inhibitor of nicotinamide phosphoribosyltransferase, represents a novel mechanism for induction of tumor cell apoptosis. Cancer Res. (2003) 63:7436–42. 14612543

[160] Olesen UH Christensen MK Bjorkling F Jaattela M Jensen PB Sehested M. Anticancer agent CHS-828 inhibits cellular synthesis of NAD. Biochem Biophys Res Commun. (2008) 367:799–804. 10.1016/j.bbrc.2008.01.019 18201551

[161] Audrito V Manago A Gaudino F Deaglio S. Targeting metabolic reprogramming in metastatic melanoma: the key role of nicotinamide phosphoribosyltransferase. (NAMPT). Semin Cell Dev Biol. (2019). 10.1016/j.semcdb.2019.05.001. [Epub ahead of print]. 31059816

[162] Howard M Grimaldi JC Bazan JF Lund FE Santos-Argumedo L Parkhouse RM. Formation and hydrolysis of cyclic ADP-ribose catalyzed by lymphocyte antigen CD38. Science. (1993) 262:1056–9. 10.1126/science.8235624 8235624

[163] Sauve AA Munshi C Lee HC Schramm VL. The reaction mechanism for CD38. A single intermediate is responsible for cyclization, hydrolysis, and base-exchange chemistries. Biochemistry. (1998) 37:13239–49. 10.1021/bi981248s 9748331

[164] Chini EN Chini CCS Espindola Netto JM De Oliveira GC Van Schooten W. The pharmacology of CD38/NADase: an emerging target in cancer and diseases of aging. Trends Pharmacol Sci. (2018) 39:424–36. 10.1016/j.tips.2018.02.001 29482842 PMC5885288

[165] Malavasi F Deaglio S Funaro A Ferrero E Horenstein AL Ortolan E. Evolution and function of the ADP ribosyl cyclase/CD38 gene family in physiology and pathology. Physiol Rev. (2008) 88:841–86. 10.1152/physrev.00035.2007 18626062

[166] Chini EN. CD38 as a regulator of cellular NAD: a novel potential pharmacological target for metabolic conditions. Curr Pharm Des. (2009) 15:57–63. 10.2174/138161209787185788 19149603 PMC2883294

[167] Hartman WR Pelleymounter LL Moon I Kalari K Liu M Wu TY. CD38 expression, function, and gene resequencing in a human lymphoblastoid cell line-based model system. Leuk Lymphoma. (2010) 51:1315–25. 10.3109/10428194.2010.483299 20470215 PMC2892000

[168] Aksoy P White TA Thompson M Chini EN. Regulation of intracellular levels of NAD: a novel role for CD38. Biochem Biophys Res Commun. (2006) 345:1386–92. 10.1016/j.bbrc.2006.05.042 16730329

[169] Young GS Choleris E Lund FE Kirkland JB. Decreased cADPR and increased NAD+ in the Cd38-/- mouse. Biochem Biophys Res Commun. (2006) 346:188–92. 10.1016/j.bbrc.2006.05.100 16750163

[170] Grozio A Sociali G Sturla L Caffa I Soncini D Salis A. CD73 protein as a source of extracellular precursors for sustained NAD+ biosynthesis in FK866-treated tumor cells. J Biol Chem. (2013) 288:25938–49. 10.1074/jbc.M113.470435 23880765 PMC3764798

[171] Camacho-Pereira J Tarrago MG Chini CCS Nin V Escande C Warner GM. CD38 dictates age-related NAD decline and mitochondrial dysfunction through an SIRT3-dependent mechanism. Cell Metab. (2016) 23:1127–39. 10.1016/j.cmet.2016.05.006 27304511 PMC4911708

[172] Malavasi F Deaglio S Damle R Cutrona G Ferrarini M Chiorazzi N. CD38 and chronic lymphocytic leukemia: a decade later. Blood. (2011) 118:3470–8. 10.1182/blood-2011-06-275610 21765022 PMC3574275

[173] Chatterjee S Daenthanasanmak A Chakraborty P Wyatt MW Dhar P Selvam SP. CD38-NAD(+)axis regulates immunotherapeutic anti-tumor T cell response. Cell Metab. (2018) 27:85–100.e108. 10.1016/j.cmet.2017.10.006 29129787 PMC5837048

[174] Kaelin WG Jr. Mcknight SL. Influence of metabolism on epigenetics and disease. Cell. (2013) 153:56–69. 10.1016/j.cell.2013.03.004 23540690 PMC3775362

[175] Dugnani E Pasquale V Bordignon C Canu A Piemonti L Monti P. Integrating T cell metabolism in cancer immunotherapy. Cancer Lett. (2017) 411:12–8. 10.1016/j.canlet.2017.09.039 28974448

[176] Buck MD O'sullivan D Pearce EL. T cell metabolism drives immunity. J Exp Med. (2015) 212:1345–60. 10.1084/jem.20151159 26261266 PMC4548052

[177] Williams MA Bevan MJ. Effector and memory CTL differentiation. Annu Rev Immunol. (2007) 25:171–92. 10.1146/annurev.immunol.25.022106.141548 17129182

[178] Sinclair LV Rolf J Emslie E Shi YB Taylor PM Cantrell DA. Control of amino-acid transport by antigen receptors coordinates the metabolic reprogramming essential for T cell differentiation. Nat Immunol. (2013) 14:500–8. 10.1038/ni.2556 23525088 PMC3672957

[179] Chang CH Qiu J O'sullivan D Buck MD Noguchi T Curtis JD. Metabolic competition in the tumor microenvironment is a driver of cancer progression. Cell. (2015) 162:1229–41. 10.1016/j.cell.2015.08.016 26321679 PMC4864363

[180] Sukumar M Roychoudhuri R Restifo NP. Nutrient competition: a new axis of tumor immunosuppression. Cell. (2015) 162:1206–8. 10.1016/j.cell.2015.08.064 26359979 PMC6327313

[181] Chang CH Pearce EL. Emerging concepts of T cell metabolism as a target of immunotherapy. Nat Immunol. (2016) 17:364–8. 10.1038/ni.3415 27002844 PMC4990080

[182] Pearce EL Poffenberger MC Chang CH Jones RG. Fueling immunity: insights into metabolism and lymphocyte function. Science. (2013) 342:1242454. 10.1126/science.1242454 24115444 PMC4486656

[183] Feske S. Calcium signalling in lymphocyte activation and disease. Nat Rev Immunol. (2007) 7:690–702. 10.1038/nri2152 17703229

[184] Clapham DE. Calcium signaling. Cell. (2007) 131:1047–58. 10.1016/j.cell.2007.11.028 18083096

[185] Ouyang K Leandro Gomez-Amaro R Stachura DL Tang H Peng X Fang X. Loss of IP3R-dependent Ca2+ signalling in thymocytes leads to aberrant development and acute lymphoblastic leukemia. Nat Commun. (2014) 5:4814. 10.1038/ncomms5814 25215520 PMC5537137

[186] Cui C Merritt R Fu L Pan Z. Targeting calcium signaling in cancer therapy. Acta Pharm Sin B. (2017) 7:3–17. 10.1016/j.apsb.2016.11.001 28119804 PMC5237760

[187] Zhang T Kraus WL. SIRT1-dependent regulation of chromatin and transcription: linking NAD(+) metabolism and signaling to the control of cellular functions. Biochim Biophys Acta. (2010) 1804:1666–75. 10.1016/j.bbapap.2009.10.022 19879981 PMC2886162

[188] Jeng MY Hull PA Fei M Kwon HS Tsou CL Kasler H. Metabolic reprogramming of human CD8(+) memory T cells through loss of SIRT1. J Exp Med. (2018) 215:51–62. 10.1084/jem.20161066 29191913 PMC5748845

[189] Feng X Zhang L Acharya C An G Wen K Qiu L. Targeting CD38 suppresses induction and function of T regulatory cells to mitigate immunosuppression in multiple myeloma. Clin Cancer Res. (2017) 23:4290–300. 10.1158/1078-0432.CCR-16-3192 28249894 PMC5540790

[190] Sica A Strauss L Consonni FM Travelli C Genazzani A Porta C. Metabolic regulation of suppressive myeloid cells in cancer. Cytokine Growth Factor Rev. (2017) 35:27–35. 10.1016/j.cytogfr.2017.05.002 28499577

[191] Franssen LE Mutis T Lokhorst HM Van De Donk N. Immunotherapy in myeloma: how far have we come? Ther Adv Hematol. (2019) 10:2040620718822660. 10.1177/2040620718822660 30719268 PMC6348514

[192] Naik J Themeli M De Jong-Korlaar R Ruiter RWJ Poddighe PJ Yuan H. CD38 as a therapeutic target for adult acute myeloid leukemia and T-cell acute lymphoblastic leukemia. Haematologica. (2019) 104:e100–103. 10.3324/haematol.2018.192757 30190344 PMC6395314

[193] Bailey SM Udoh US Young ME. Circadian regulation of metabolism. J Endocrinol. (2014) 222:R75–96. 10.1530/JOE-14-0200 24928941 PMC4109003

[194] Al-Khami AA Mehrotra S Nishimura MI. Adoptive immunotherapy of cancer: gene transfer of T cell specificity. Self Nonself. (2011) 2:80–4. 10.4161/self.2.2.15832 22299059 PMC3268993

[195] Rosenberg SA Restifo NP. Adoptive cell transfer as personalized immunotherapy for human cancer. Science. (2015) 348:62–8. 10.1126/science.aaa4967 25838374 PMC6295668

[196] Paulos CM Carpenito C Plesa G Suhoski MM Varela-Rohena A Golovina TN. The inducible costimulator. (ICOS) is critical for the development of human T(H)17 cells. Sci Transl Med. (2010) 2:55ra78. 10.1126/scitranslmed.3000448 20980695 PMC6282816

[197] Muranski P Borman ZA Kerkar SP Klebanoff CA Ji Y Sanchez-Perez L. Th17 cells are long lived and retain a stem cell-like molecular signature. Immunity. (2011) 35:972–85. 10.1016/j.immuni.2011.09.019 22177921 PMC3246082

[198] Chen L Diao L Yang Y Yi X Rodriguez BL Li Y. CD38-mediated immunosuppression as a mechanism of tumor cell escape from PD-1/PD-L1 blockade. Cancer Discov. (2018) 8:1156–75. 10.1158/2159-8290.CD-17-1033 30012853 PMC6205194

[199] Rine J Herskowitz I. Four genes responsible for a position effect on expression from HML and HMR in Saccharomyces cerevisiae. Genetics. (1987) 116:9–22. 3297920 10.1093/genetics/116.1.9 PMC1203125

[200] Frye RA. Phylogenetic classification of prokaryotic and eukaryotic Sir2-like proteins. Biochem Biophys Res Commun. (2000) 273:793–8. 10.1006/bbrc.2000.3000 10873683

[201] Michishita E Park JY Burneskis JM Barrett JC Horikawa I. Evolutionarily conserved and nonconserved cellular localizations and functions of human SIRT proteins. Mol Biol Cell. (2005) 16:4623–35. 10.1091/mbc.e05-01-0033 16079181 PMC1237069

[202] North BJ Verdin E. Interphase nucleo-cytoplasmic shuttling and localization of SIRT2 during mitosis. PLoS ONE. (2007) 2:e784. 10.1371/journal.pone.0000784 17726514 PMC1949146

[203] Tanno M Kuno A Yano T Miura T Hisahara S Ishikawa S. Induction of manganese superoxide dismutase by nuclear translocation and activation of SIRT1 promotes cell survival in chronic heart failure. J Biol Chem. (2010) 285:8375–82. 10.1074/jbc.M109.090266 20089851 PMC2832987

[204] Iwahara T Bonasio R Narendra V Reinberg D. SIRT3 functions in the nucleus in the control of stress-related gene expression. Mol Cell Biol. (2012) 32:5022–34. 10.1128/MCB.00822-12 23045395 PMC3510539

[205] Yanagisawa S Baker JR Vuppusetty C Koga T Colley T Fenwick P. The dynamic shuttling of SIRT1 between cytoplasm and nuclei in bronchial epithelial cells by single and repeated cigarette smoke exposure. PLoS ONE. (2018) 13:e0193921. 10.1371/journal.pone.0193921 29509781 PMC5839577

[206] Sauve AA Wolberger C Schramm VL Boeke JD. The biochemistry of sirtuins. Annu Rev Biochem. (2006) 75:435–65. 10.1146/annurev.biochem.74.082803.133500 16756498

[207] Bheda P Jing H Wolberger C Lin H. The substrate specificity of sirtuins. Annu Rev Biochem. (2016) 85:405–29. 10.1146/annurev-biochem-060815-014537 27088879

[208] Haigis MC Sinclair DA. Mammalian sirtuins: biological insights and disease relevance. Annu Rev Pathol. (2010) 5:253–95. 10.1146/annurev.pathol.4.110807.092250 20078221 PMC2866163

[209] Finkel T Deng CX Mostoslavsky R. Recent progress in the biology and physiology of sirtuins. Nature. (2009) 460:587–91. 10.1038/nature08197 19641587 PMC3727385

[210] Houtkooper RH Pirinen E Auwerx J. Sirtuins as regulators of metabolism and healthspan. Nat Rev Mol Cell Biol. (2012) 13:225–38. 10.1038/nrm3293 22395773 PMC4872805

[211] Natoli G. When sirtuins and NF-kappaB collide. Cell. (2009) 136:19–21. 10.1016/j.cell.2008.12.034 19135883

[212] Preyat N Leo O. Sirtuin deacylases: a molecular link between metabolism and immunity. J Leukoc Biol. (2013) 93:669–80. 10.1189/jlb.1112557 23325925

[213] Vachharajani VT Liu T Wang X Hoth JJ Yoza BK Mccall CE. Sirtuins link inflammation and metabolism. J Immunol Res. (2016) 2016:8167273. 10.1155/2016/8167273 26904696 PMC4745579

[214] Verdin E. The many faces of sirtuins: Coupling of NAD metabolism, sirtuins and lifespan. Nat Med. (2014) 20:25–7. 10.1038/nm.3447 24398962

[215] Chen X Lu Y Zhang Z Wang J Yang H Liu G. Intercellular interplay between Sirt1 signalling and cell metabolism in immune cell biology. Immunology. (2015) 145:455–67. 10.1111/imm.12473 25890999 PMC4515126

[216] Reiner SL Epigenetic control in the immune response. Hum Mol Genet. (2005) 14 Spec 1:R41–6. 10.1093/hmg/ddi115 15809272

[217] Busslinger M Tarakhovsky A. Epigenetic control of immunity. Cold Spring Harb Perspect Biol. (2014) 6:a024174. 10.1101/cshperspect.a024174 24890513 PMC4031963

[218] Jasiulionis MG. Abnormal epigenetic regulation of immune system during aging. Front Immunol. (2018) 9:197. 10.3389/fimmu.2018.00197 29483913 PMC5816044

[219] Jaenisch R Bird A. Epigenetic regulation of gene expression: how the genome integrates intrinsic and environmental signals. Nat Genet. (2003) 33:245–54. 10.1038/ng1089 12610534

[220] Glass CK Natoli G. Molecular control of activation and priming in macrophages. Nat Immunol. (2016) 17:26–33. 10.1038/ni.3306 26681459 PMC4795476

[221] O'neill LA Pearce EJ. Immunometabolism governs dendritic cell and macrophage function. J Exp Med. (2016) 213:15–23. 10.1084/jem.20151570 26694970 PMC4710204

[222] Rodriguez-Prados JC Traves PG Cuenca J Rico D Aragones J Martin-Sanz P. Substrate fate in activated macrophages: a comparison between innate, classic, and alternative activation. J Immunol. (2010) 185:605–14. 10.4049/jimmunol.0901698 20498354

[223] Wang X Buechler NL Woodruff AG Long DL Zabalawi M Yoza BK. Sirtuins and immuno-metabolism of sepsis. Int J Mol Sci. (2018) 19:2738. 10.3390/ijms19092738 30216989 PMC6164482

[224] Rodgers JT Lerin C Haas W Gygi SP Spiegelman BM Puigserver P. Nutrient control of glucose homeostasis through a complex of PGC-1alpha and SIRT1. Nature. (2005) 434:113–8. 10.1038/nature03354 15744310

[225] Canto C Auwerx J. PGC-1alpha, SIRT1 and AMPK, an energy sensing network that controls energy expenditure. Curr Opin Lipidol. (2009) 20:98–105. 10.1097/MOL.0b013e328328d0a4 19276888 PMC3627054

[226] Canto C Gerhart-Hines Z Feige JN Lagouge M Noriega L Milne JC. AMPK regulates energy expenditure by modulating NAD+ metabolism and SIRT1 activity. Nature. (2009) 458:1056–60. 10.1038/nature07813 19262508 PMC3616311

[227] Simmons GE Pruitt WM Pruitt K. Diverse roles of SIRT1 in cancer biology and lipid metabolism. Int J Mol Sci. (2015) 16:950–65. 10.3390/ijms16010950 25569080 PMC4307284

[228] Kauppinen A Suuronen T Ojala J Kaarniranta K Salminen A. Antagonistic crosstalk between NF-kappaB and SIRT1 in the regulation of inflammation and metabolic disorders. Cell Signal. (2013) 25:1939–48. 10.1016/j.cellsig.2013.06.007 23770291

[229] Salminen A Kaarniranta K Kauppinen A. Crosstalk between oxidative stress and SIRT1: impact on the aging process. Int J Mol Sci. (2013) 14:3834–59. 10.3390/ijms14023834 23434668 PMC3588074

[230] Yeung F Hoberg JE Ramsey CS Keller MD Jones DR Frye RA. Modulation of NF-kappaB-dependent transcription and cell survival by the SIRT1 deacetylase. EMBO J. (2004) 23:2369–80. 10.1038/sj.emboj.7600244 15152190 PMC423286

[231] Yang H Zhang W Pan H Feldser HG Lainez E Miller C. SIRT1 activators suppress inflammatory responses through promotion of p65 deacetylation and inhibition of NF-kappaB activity. PLoS ONE. (2012) 7:e46364. 10.1371/journal.pone.0046364 23029496 PMC3460821

[232] Schug TT Xu Q Gao H Peres-Da-Silva A Draper DW Fessler MB. Myeloid deletion of SIRT1 induces inflammatory signaling in response to environmental stress. Mol Cell Biol. (2010) 30:4712–21. 10.1128/MCB.00657-10 20647536 PMC2950528

[233] Leiser SF Kaeberlein M. A role for SIRT1 in the hypoxic response. Mol Cell. (2010) 38:779–80. 10.1016/j.molcel.2010.06.015 20620950

[234] Lim JH Lee YM Chun YS Chen J Kim JE Park JW. Sirtuin 1 modulates cellular responses to hypoxia by deacetylating hypoxia-inducible factor 1alpha. Mol Cell. (2010) 38:864–78. 10.1016/j.molcel.2010.05.023 20620956

[235] Joo HY Yun M Jeong J Park ER Shin HJ Woo SR SIRT1 deacetylates and stabilizes hypoxia-inducible factor-α. (HIF-1α) via direct interactions during hypoxia. Biochem Biophys Res Commun. (2015) 462:294–300. 10.1016/j.bbrc.2015.04.119 25979359

[236] Yu Q Dong L Li Y Liu G. SIRT1 and HIF1α signaling in metabolism and immune responses. Cancer Lett. (2018) 418:20–6. 10.1016/j.canlet.2017.12.035 29306019

[237] Liu G Bi Y Shen B Yang H Zhang Y Wang X. SIRT1 limits the function and fate of myeloid-derived suppressor cells in tumors by orchestrating HIF-1α-dependent glycolysis. Cancer Res. (2014) 74:727–37. 10.1158/0008-5472.CAN-13-2584 24351289

[238] Wang Y Bi Y Chen X Li C Li Y Zhang Z. Histone deacetylase SIRT1 negatively regulates the differentiation of interleukin-9-producing CD4(+) T cells. Immunity. (2016) 44:1337–49. 10.1016/j.immuni.2016.05.009 27317260

[239] Chalkiadaki A Guarente L. The multifaceted functions of sirtuins in cancer. Nat Rev Cancer. (2015) 15:608–24. 10.1038/nrc3985 26383140

[240] Henning AN Roychoudhuri R Restifo NP. Epigenetic control of CD8(+) T cell differentiation. Nat Rev Immunol. (2018) 18:340–56. 10.1038/nri.2017.146 29379213 PMC6327307

[241] Zhang X Liu J Cao X. Metabolic control of T-cell immunity via epigenetic mechanisms. Cell Mol Immunol. (2018) 15:203–5. 10.1038/cmi.2017.115 29082922 PMC5843618

[242] Legutko A Marichal T Fievez L Bedoret D Mayer A De Vries H. Sirtuin 1 promotes Th2 responses and airway allergy by repressing peroxisome proliferator-activated receptor-γ activity in dendritic cells. J Immunol. (2011) 187:4517–29. 10.4049/jimmunol.1101493 21948987

[243] Zhang J Lee SM Shannon S Gao B Chen W Chen A. The type III histone deacetylase Sirt1 is essential for maintenance of T cell tolerance in mice. J Clin Invest. (2009) 119:3048–58. 10.1172/JCI38902 19729833 PMC2752073

[244] Kuroda S Yamazaki M Abe M Sakimura K Takayanagi H Iwai Y. Basic leucine zipper transcription factor, ATF-like. (BATF) regulates epigenetically and energetically effector CD8 T-cell differentiation via Sirt1 expression. Proc Natl Acad Sci USA. (2011) 108:14885–9. 10.1073/pnas.1105133108 21873234 PMC3169148

[245] Han MK Song EK Guo Y Ou X Mantel C Broxmeyer HE. SIRT1 regulates apoptosis and Nanog expression in mouse embryonic stem cells by controlling p53 subcellular localization. Cell Stem Cell. (2008) 2:241–51. 10.1016/j.stem.2008.01.002 18371449 PMC2819008

[246] Boutant M Canto C. SIRT1 metabolic actions: integrating recent advances from mouse models. Mol Metab. (2014) 3:5–18. 10.1016/j.molmet.2013.10.006 24567900 PMC3929913

[247] Imperatore F Maurizio J Vargas Aguilar S Busch CJ Favret J Kowenz-Leutz E. SIRT1 regulates macrophage self-renewal. EMBO J. (2017) 36:2353–72. 10.15252/embj.201695737 28701484 PMC5556267

[248] Gomes P Fleming Outeiro T Cavadas C. Emerging role of sirtuin 2 in the regulation of mammalian metabolism. Trends Pharmacol Sci. (2015) 36:756–68. 10.1016/j.tips.2015.08.001 26538315

[249] Chadha S Wang L Hancock WW Beier UH. Sirtuin-1 in immunotherapy: a Janus-headed target. J Leukoc Biol. (2019). 10.1002/JLB.2RU1118-422R 30605226 PMC7477756

[250] Beier UH Wang L Bhatti TR Liu Y Han R Ge G. Sirtuin-1 targeting promotes Foxp3+ T-regulatory cell function and prolongs allograft survival. Mol Cell Biol. (2011) 31:1022–9. 10.1128/MCB.01206-10 21199917 PMC3067815

[251] Daenthanasanmak A Iamsawat S Chakraborty P Nguyen HD Bastian D Liu C. Targeting Sirt-1 controls GVHD by inhibiting T-cell allo-response and promoting Treg stability in mice. Blood. (2019) 133:266–79. 10.1182/blood-2018-07-863233 30514750 PMC6337874

[252] Liu TF Mccall CE. Deacetylation by SIRT1 reprograms inflammation and cancer. Genes Cancer. (2013) 4:135–47. 10.1177/1947601913476948 24020005 PMC3764465

[253] Bitterman KJ Anderson RM Cohen HY Latorre-Esteves M Sinclair DA. Inhibition of silencing and accelerated aging by nicotinamide, a putative negative regulator of yeast sir2 and human SIRT1. J Biol Chem. (2002) 277:45099–107. 10.1074/jbc.M205670200 12297502

[254] Audrito V Vaisitti T Rossi D Gottardi D D'arena G Laurenti L. Nicotinamide blocks proliferation and induces apoptosis of chronic lymphocytic leukemia cells through activation of the p53/miR-34a/SIRT1 tumor suppressor network. Cancer Res. (2011) 71:4473–83. 10.1158/0008-5472.CAN-10-4452 21565980

[255] Revollo JR Grimm AA Imai S. The NAD biosynthesis pathway mediated by nicotinamide phosphoribosyltransferase regulates Sir2 activity in mammalian cells. J Biol Chem. (2004) 279:50754–63. 10.1074/jbc.M408388200 15381699

[256] Geng J Liu A. Heme-dependent dioxygenases in tryptophan oxidation. Arch Biochem Biophys. (2014) 544:18–26. 10.1016/j.abb.2013.11.009 24295960

[257] Munn DH Zhou M Attwood JT Bondarev I Conway SJ Marshall B. Prevention of allogeneic fetal rejection by tryptophan catabolism. Science. (1998) 281:1191–3. 10.1126/science.281.5380.1191 9712583

[258] Mbongue JC Nicholas DA Torrez TW Kim NS Firek AF Langridge WH. The role of indoleamine 2, 3-dioxygenase in immune suppression and autoimmunity. Vaccines. (2015) 3:703–29. 10.3390/vaccines3030703 26378585 PMC4586474

[259] Takikawa O. Biochemical and medical aspects of the indoleamine 2,3-dioxygenase-initiated L-tryptophan metabolism. Biochem Biophys Res Commun. (2005) 338:12–9. 10.1016/j.bbrc.2005.09.032 16176799

[260] King NJ Thomas SR. Molecules in focus: indoleamine 2,3-dioxygenase. Int J Biochem Cell Biol. (2007) 39:2167–72. 10.1016/j.biocel.2007.01.004 17320464

[261] Katz JB Muller AJ Prendergast GC. Indoleamine 2,3-dioxygenase in T-cell tolerance and tumoral immune escape. Immunol Rev. (2008) 222:206–21. 10.1111/j.1600-065X.2008.00610.x 18364004

[262] Puccetti P Grohmann U. IDO and regulatory T cells: a role for reverse signalling and non-canonical NF-kappaB activation. Nat Rev Immunol. (2007) 7:817–23. 10.1038/nri2163 17767193

[263] Mellor AL Munn DH. IDO expression by dendritic cells: tolerance and tryptophan catabolism. Nat Rev Immunol. (2004) 4:762–74. 10.1038/nri1457 15459668

[264] Munn DH Mellor AL. Indoleamine 2,3 dioxygenase and metabolic control of immune responses. Trends Immunol. (2013) 34:137–43. 10.1016/j.it.2012.10.001 23103127 PMC3594632

[265] Munn DH Mellor AL. IDO in the tumor microenvironment: inflammation, counter-regulation, and tolerance. Trends Immunol. (2016) 37:193–207. 10.1016/j.it.2016.01.002 26839260 PMC4916957

[266] Platten M Wick W Van Den Eynde BJ. Tryptophan catabolism in cancer: beyond IDO and tryptophan depletion. Cancer Res. (2012) 72:5435–40. 10.1158/0008-5472.CAN-12-0569 23090118

[267] Opitz CA Litzenburger UM Sahm F Ott M Tritschler I Trump S. An endogenous tumour-promoting ligand of the human aryl hydrocarbon receptor. Nature. (2011) 478:197–203. 10.1038/nature10491 21976023

[268] Uyttenhove C Pilotte L Theate I Stroobant V Colau D Parmentier N. Evidence for a tumoral immune resistance mechanism based on tryptophan degradation by indoleamine 2,3-dioxygenase. Nat Med. (2003) 9:1269–74. 10.1038/nm934 14502282

[269] Hornyak L Dobos N Koncz G Karanyi Z Pall D Szabo Z. The role of indoleamine-2,3-dioxygenase in cancer development, diagnostics, and therapy. Front Immunol. (2018) 9:151. 10.3389/fimmu.2018.00151 29445380 PMC5797779

[270] Yu CP Fu SF Chen X Ye J Ye Y Kong LD. The clinicopathological and prognostic significance of IDO1 expression in human solid tumors: evidence from a systematic review and meta-analysis. Cell Physiol Biochem. (2018) 49:134–43. 10.1159/000492849 30134237

[271] Zhao Q Kuang DM Wu Y Xiao X Li XF Li TJ. Activated CD69+ T cells foster immune privilege by regulating IDO expression in tumor-associated macrophages. J Immunol. (2012) 188:1117–24. 10.4049/jimmunol.1100164 22184722

[272] Pinton L Solito S Damuzzo V Francescato S Pozzuoli A Berizzi A. Activated T cells sustain myeloid-derived suppressor cell-mediated immune suppression. Oncotarget. (2016) 7:1168–84. 10.18632/oncotarget.6662 26700461 PMC4811451

[273] Lob S Konigsrainer A Rammensee HG Opelz G Terness P. Inhibitors of indoleamine-2,3-dioxygenase for cancer therapy: can we see the wood for the trees? Nat Rev Cancer. (2009) 9:445–52. 10.1038/nrc2639 19461669

[274] Brochez L Chevolet I Kruse V. The rationale of indoleamine 2,3-dioxygenase inhibition for cancer therapy. Eur J Cancer. (2017) 76:167–82. 10.1016/j.ejca.2017.01.011 28324751

[275] Prendergast GC Malachowski WP Duhadaway JB Muller AJ. Discovery of IDO1 inhibitors: from bench to bedside. Cancer Res. (2017) 77:6795–811. 10.1158/0008-5472.CAN-17-2285 29247038 PMC6021761

[276] Hou DY Muller AJ Sharma MD Duhadaway J Banerjee T Johnson M. Inhibition of indoleamine 2,3-dioxygenase in dendritic cells by stereoisomers of 1-methyl-tryptophan correlates with antitumor responses. Cancer Res. (2007) 67:792–801. 10.1158/0008-5472.CAN-06-2925 17234791

[277] Holmgaard RB Zamarin D Munn DH Wolchok JD Allison JP. Indoleamine 2,3-dioxygenase is a critical resistance mechanism in antitumor T cell immunotherapy targeting CTLA-4. J Exp Med. (2013) 210:1389–402. 10.1084/jem.20130066 23752227 PMC3698523

